# Intrusion Detection Datasets for IIoT and ICS: A Taxonomic Review with a Decision-Aid Scoring Rubric

**DOI:** 10.3390/s26134099

**Published:** 2026-06-27

**Authors:** Ayman Termanini, Hadj Bourdoucen, Dawood Al-Abri, Ahmed Al Maashri

**Affiliations:** Department of Electrical and Computer Engineering, Sultan Qaboos University, Al Khodh 123, Oman; s132237@student.squ.edu.om (A.T.); alabrid@squ.edu.om (D.A.-A.); amaashari@squ.edu.om (A.A.M.)

**Keywords:** CPS/IIoT security, ICS security, OT intrusion detection, dataset, MITRE ATT&CK, anomaly detection, machine learning for security, AI

## Abstract

Dataset quality significantly affects the effectiveness of a machine learning (ML) model in an intrusion detection system (IDS) for cyber-physical industrial control systems (CPS/ICS) and Industrial Internet of Things (IIoT). Existing surveys compare datasets qualitatively or along limited dimensions, whereas this review introduces quantitative documentation and decision-aid scoring across 23 ICS/OT/IIoT datasets. These datasets are analyzed along seven measurable axes, with their attacks mapped to MITRE ATT&CK for ICS tactics. Quantitatively, 14 of the 23 datasets (60.9%) are built on physical testbeds, and 22 of the 23 map to MITRE ATT&CK for ICS, spanning 11 of the 12 tactics. We introduce a checklist for documentation completeness (0–7) and a decision-aid rubric (0–15) covering realism, attack diversity, class imbalance, documentation, and reproducibility. Protocol coverage across these datasets is skewed toward Modbus (13 of 23 datasets, 57%), while many other protocols (such as Profinet and OPC UA) are underrepresented relative to their industry deployment. The available datasets show structural gaps in capturing multi-stage adversary behavior. In practice, dataset selection should pair a realism-anchored dataset with a high-reproducibility one, and account for protocol diversity and APT representation.

## 1. Introduction

**Figure 1:**
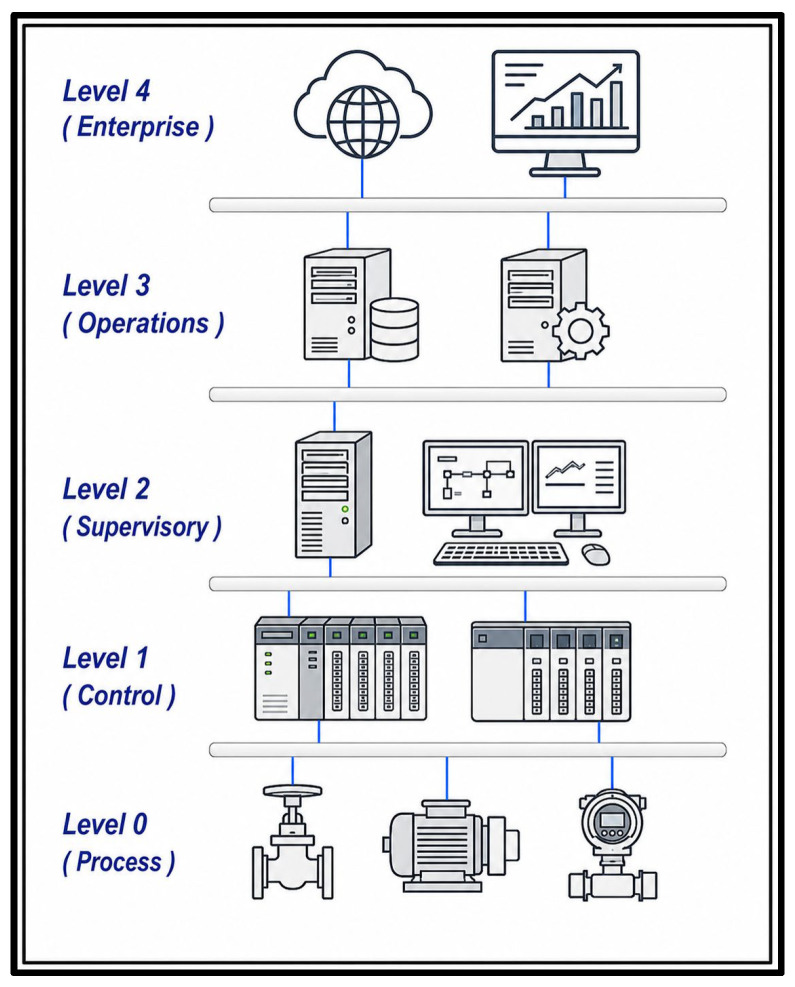
Standard ICS interfacing diagram.

Industrial Control Systems (ICS) are core components of critical infrastructure, used across many sectors to control operations through automation. They combine several technologies, such as SCADA (Supervisory Control and Data Acquisition) systems, PLCs, and field devices. ICS networks provide the infrastructure that links these components, allowing real-time monitoring and control of industrial plants. The typical architecture of an industrial network, following the Purdue Reference Model (Figure 1), comprises five levels, from the process layer up to the enterprise layer. The Industrial Internet of Things (IIoT) extends ICS with additional heterogeneity in connectivity, vendors, protocols, and cloud-based services. The convergence of IT and OT (Operational Technology) environments and the adoption of IIoT have widened the cyber-attack surface of industrial environments [1].

The Stuxnet attack [2] was one of the first warnings of the damage cyber threats can inflict on industrial systems, illustrating that the consequences can extend to real-world physical damage. Successful cyber-attacks on ICS can disrupt industrial plants and can even lead to loss of human life, a serious concern for industries and governments [3]. The frequency and complexity of attacks on critical infrastructure require security strategies that combine technical defenses with organizational practices and operator training. IDS is one such solution that detects potential threats to the network by inspecting and analyzing network traffic. It can be deployed as a software solution or as a dedicated hardware appliance.

**Figure 2:**
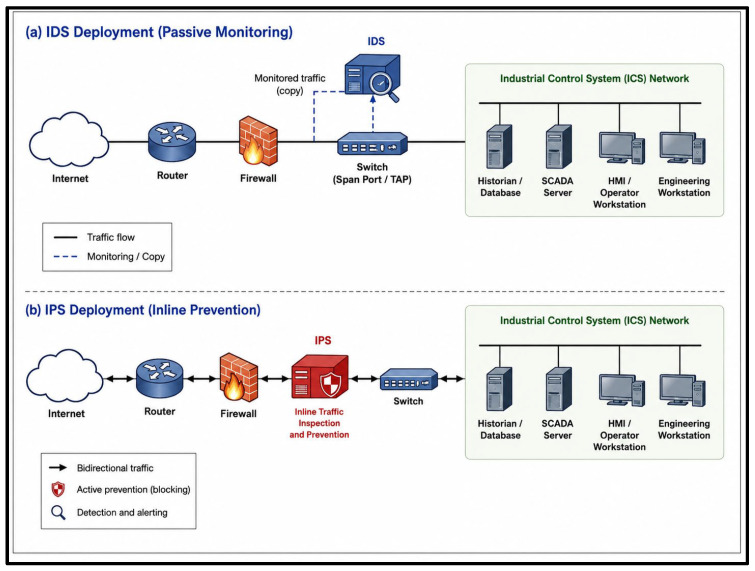
Intrusion detection/prevention deployments.

Figure 2 shows two IDS deployments; when installed in line with the traffic, the system can also control what passes, a configuration known as an intrusion prevention system (IPS). There are two main IDS mechanisms. One is signature-based, which compares network packets against a known database of malicious traffic, while the other is anomaly-based, which builds a baseline of normal network traffic and inspects for any deviations. ML approaches are receiving growing attention in IDS technologies, in both industry and academia.

Two factors explain the recent uptake of ML-based IDS for ICS: an increasing threat volume against ICS infrastructure, and the lowered engineering barrier provided by open-source ML frameworks (Scikit-learn, PyTorch, TensorFlow) and pre-trained models. Three ML families are used in ICS-IDS research, each with different dataset requirements. Supervised learning involves training a classifier using labeled data of normal and attack instances. It achieves good detection accuracy on the dataset it is trained on, but generalizes poorly to new datasets and attacks. This type is limited by the quality of the datasets. Most of the ICS-IDS research in the literature is based on supervised techniques (Random Forest, SVM, deep neural networks). Unsupervised learning requires no labels and is promising for ICS applications where labeled instances are scarce, but it is sensitive to non-malicious operational changes and is prone to high false-positive rates. Semi-supervised learning uses limited labeled data, while the majority of data is unlabeled. It combines the advantages of both approaches and is of growing interest due to the high labeling cost [4,5,6].

Datasets shape three stages of the ML pipeline: training, validation, and testing. Training sets are used to fit model parameters by minimizing the loss between predictions and labels. Validation sets are held out during training and used to tune hyperparameters; they expose overfitting before the model meets unseen data. Test sets, used only after training is complete, provide the unbiased performance estimate that will generalize to deployment. The three stages are interdependent: poor partitioning of any one undermines the others. Dataset quality, therefore, propagates into every downstream IDS performance metric [7].

To structure the analysis, this review is guided by four research questions: RQ1. Which ICS/IIoT intrusion-detection datasets are available through Q1 2026, and how can they be characterized along measurable taxonomic dimensions? RQ2. How completely are these datasets documented, and how reproducible are they for IDS evaluation? RQ3. What adversary behaviors do their attacks cover when mapped to MITRE ATT&CK for ICS, and where are the coverage gaps? RQ4. Which datasets are most suitable for specific IDS evaluation use cases, and how robust is that ranking?

This study offers a multi-axis taxonomic analysis of cybersecurity datasets for ICS network intrusion detection. A seven-dimensional taxonomy is applied to 23 ICS-specific datasets, and a quantitative documentation-completeness score is used in place of the qualitative framing adopted by prior surveys. A five-criterion decision-aid scoring rubric is proposed and yields per-research-direction dataset recommendations. The rest of the paper is organized as follows: Section 2 reviews related work in the ICS IDS literature; Section 3 describes the methodology used to study and analyze the datasets; Section 4 surveys the selected datasets; Section 5 analyzes the datasets according to the seven taxonomic dimensions; Section 6 presents the results and findings, including the decision-aid scoring rubric; Section 7 discusses future research directions; and Section 8 concludes the paper.

## 2. Related Work

Conti et al. [1] examined a large number of testbeds and datasets and verified the performance of the algorithms on them to provide a baseline. The period covered by our paper is extended to Q1 2026, whereas Conti et al. cover up to 2020. We introduce an explicit per-dataset documentation-completeness scoring, and add a use-case ranking rubric.

The protocol and feature taxonomy presented here is complementary to the taxonomy of attack paths used by Choi et al. [8] for comparing ICS datasets. Mubarak et al. [9] presented an ICS testbed and ML attack-detection results but did not provide a taxonomic analysis. Mitseva et al. [10] investigated representativeness properties and dataset constraints in a limited way. Both works compare datasets as objects, but do not provide quantitative documentation, scoring, or per-use-case rankings. Pinto et al. [6] conducted a survey of ML-based IDS for critical infrastructure, which also included the datasets used to train the ML-based IDS, whereas our paper focuses on ICS-related datasets. Koay et al. [5] conducted a survey of ML in ICS security and found that dataset diversity is an open challenge. Dehlaghi-Ghadim et al. [11] point out that the common datasets do not contain realistic ICS network data, which limits the evaluation of ML algorithms for ICS-IDS. Hu et al. [12] likewise point out that another challenge for IDS methodologies is the lack of comprehensive and realistic datasets that accurately reflect real ICS network traffic.

The quality of the data has a significant impact on the feature engineering performed in the pre-processing phase of model training. Anwar et al. [13] discuss attribute extension to improve anomaly detection in SCADA networks. They conclude that the ability of ML algorithms to detect attacks can be greatly improved by modifying and extending the dataset attributes. Sun et al. [14] propose a hybrid ML model to overcome the high dimensionality and class imbalance issues frequently encountered in ICS datasets by using feature selection. Their approach shows that the use of different ML techniques in conjunction with good feature selection can result in more powerful and reliable IDS in ICS environments.

Yang et al. [15] systematically reviewed anomaly-based network intrusion detection methods and datasets, without the ICS-process focus adopted here; Kheddar et al. [16] surveyed deep transfer learning for ICS-IDS, with dataset coverage as a secondary axis. Martins et al. [17] systematically reviewed adversarial machine learning in intrusion- and malware-detection scenarios, addressing model robustness rather than the dataset characterization that is our focus. Mubarak et al. [9] provided a focused analysis of public ICS datasets using exploratory data analysis (EDA) and a Random Forest baseline.

Across these works, several limitations recur, and Table 1 makes them explicit. Their comparisons remain qualitative or limited in dimension: none attaches a per-dataset documentation-completeness score, none offers a use-case decision rubric or per-use-case rankings, and none maps dataset attacks to a standardized adversary model such as MITRE ATT&CK for ICS. Their coverage also predates the recent wave of dataset releases, reaching 2022 at the latest, so datasets published from 2023 onward are not assessed. Existing taxonomies, therefore, describe datasets, often along coarse categories, without giving a practitioner a reproducible basis for judging how completely a dataset is documented, how reproducible it is, or which dataset best fits a given evaluation.

In addition to the taxonomies listed above, this work differs from previous surveys in three specific ways: (i) coverage to Q1 2026; (ii) the introduction of a quantitative documentation-completeness score in Section 6; and (iii) the production of per-use-case dataset rankings. Table 1 contrasts this review with recent surveys and multi-dataset studies across the dimensions that define its contribution.

## 3. Study Methodology

### 3.1. Search Strategy and Selection Procedure

We searched for datasets using structured queries in international publishers (Springer, IEEE Xplore, ScienceDirect, MDPI, and arXiv). These databases were chosen because they index the main peer-reviewed literature for ICS, SCADA, and IIoT security and dataset research across the major engineering and computer-science publishers (IEEE, Elsevier, Springer, and MDPI). The arXiv is included to capture newly released datasets not yet formally indexed. Search terms were combined using the OR operator for “intrusion detection”, “anomaly detection”, and “IDS”; the AND operator for “industrial control system”, “ICS”, “SCADA”, “cyber-physical system”, and “IIoT”; and the AND operator for “dataset”, “testbed”, and “data collection”.

Only datasets that satisfy the following criteria were retained: (a) the dataset represents ICS/OT/SCADA/IIoT network traffic or process data that is captured or simulated; (b) the dataset is documented in a peer-reviewed publication or readily accessible technical report; and (c) the dataset is publicly available or available on request, or a labeled subset is referenced in subsequent IDS research. We also included established ICS and OT benchmark datasets that are widely reused in the intrusion-detection literature, applying the same inclusion criteria. Because these are identified from the literature rather than only through the listed databases, several of their primary papers appear in other venues, such as ACM. Records were excluded when they contained IT-only traffic without OT process or industrial-protocol data. Finally, 23 datasets constitute the surviving set after these filters. Datasets containing only IT without OT traffic are mentioned for reference but excluded from the study scope. This is a narrative taxonomic review, not a systematic review; we did not run a PRISMA-style screening pipeline.

**Figure 3:**
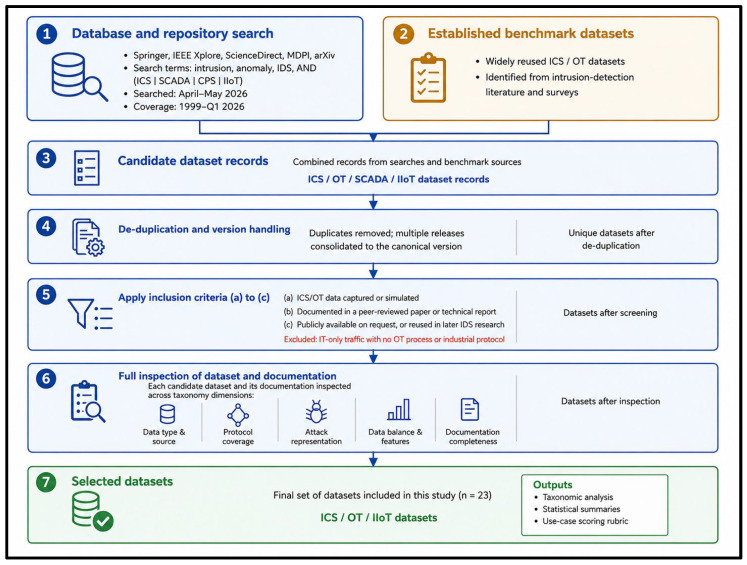
Dataset selection and screening workflow.

The 23 surviving datasets were selected by the criteria above, applied by the first author and then independently reviewed and confirmed by the co-authors. The search was carried out in April and May 2026 and covers dataset releases up to the first quarter of 2026; the databases, search terms, and inclusion and exclusion criteria stated above are reported in full so that the search can be repeated. Duplicate records were removed, and where a dataset is published in several releases, for example, the successive HAI releases, the most complete documented release was treated as the canonical entry, with material version differences noted in Section 4.3. This selection workflow is summarized in Figure 3. Because the 23 datasets are a finite, curated set of established and recent benchmark datasets assembled through this hybrid strategy rather than retrieved through large-scale database screening, a per-stage PRISMA identification-screening-exclusion record count would add little for this selection beyond the explicit criteria already reported above. The databases, search terms, inclusion and exclusion criteria, and de-duplication rule, together with the selection workflow in Figure 3, are provided to make the selection transparent and reproducible.

### 3.2. Analytical Axes

Seven measurable dimensions are considered to analyze each dataset. First, we check the environment where the dataset is captured: whether from a physical testbed, by simulation of a process model, or from a real operational network. Dataset realism is assessed from the operational realism of this environment (real operational network, then physical or hardware-in-the-loop testbed, then pure simulation) together with the provenance of the attack traffic (naturally occurring or incident-captured versus deliberately injected). It is formalized as a 0 to 3 realism score in the decision-aid rubric (Section 6.5.1). Second, we identify the data type and distinguish process data from networking traffic. Third, we highlight how the dataset is delivered (PCAP, CSV, or other file formats). Fourth, the OT protocols are checked (Modbus TCP/RTU, S7Comm, and others). Fifth, the attack representation is analyzed for the diversity of attacks. Sixth, we identify the dataset features and classes and check whether an imbalance exists between the majority class (normal traffic) and the minority anomaly class. Finally, the documentation is assessed using a novel approach, and we assign a quantitative score to each dataset using a 0–7 checklist, awarding one point when each of the following elements is documented: system architecture, protocol, attack or anomaly methodology, record or class counts, feature description, labeling or ground-truth method, and availability conditions. We then convert the documentation score into three classes, from Mild to Comprehensive. The aim is to provide a comparison of documentation quality across datasets to support a scoring approach in the decision-aid rubric. Both scoring instruments combine their elements with equal weights; the robustness of this choice is verified by a sensitivity analysis in Section 6.5.1.

### 3.3. Dataset Characteristics

This study evaluates one quality characteristic: the balance between normal and attack traffic (reported as the imbalance ratio; see Section 5.3). Examining the features in these datasets is also important, as they characterize the data and determine what patterns ML models can learn to identify. We therefore record each dataset’s feature dimensionality and distinguish networking-traffic features from process values. The protocols represented in the datasets are additionally investigated, since different industrial communication stacks expose distinct vulnerabilities and attack vectors. The various attack types are categorized to assess the comprehensiveness of each dataset’s coverage of realistic adversary behavior. We map the identified attacks to the MITRE ATT&CK for ICS tactics [18].

## 4. Dataset Survey

### 4.1. Datasets Used in ICS-IDS

Researchers in ICS-IDS initially had to rely on IT datasets for model training. Then OT-specific datasets began to emerge in the mid-2010s. We list the datasets widely used in the literature in Table 2, along with their types and publication years.

**Figure 4:**
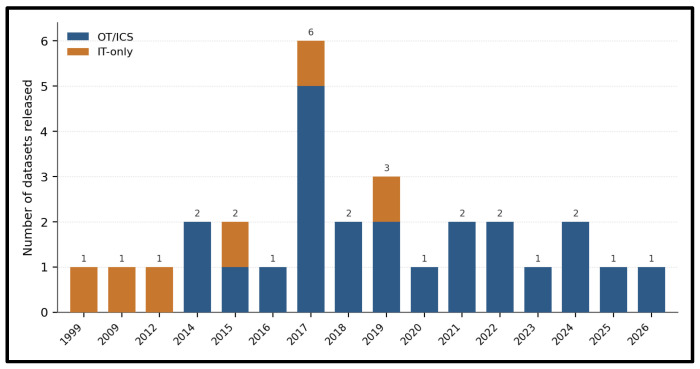
Dataset releases by year (1999–2026), grouped by OT/ICS vs. IT-only.

The temporal distribution of OT vs. IT-only dataset releases is illustrated in Figure 4. The timeline shows that ICS/IIoT dataset releases have accelerated since around 2017, with many datasets published in the last five years, reflecting the field’s growing maturity and the rising demand for ML-ready benchmarks. Recent releases such as Edge-IIoTset and ICS-NAD broaden coverage and modern IIoT/edge framings, yet the field remains young, and the number of well-documented, openly available datasets is still small, which limits large-sample statistics and any claim about long-term temporal trends.

### 4.2. Selected Datasets for This Study

Our study focuses only on datasets related to ICS network traffic or process data. Table 3 presents the selected datasets with general information that gives researchers a first overview. The industrial domain spans water, gas, electricity, and other sectors. The environment clarifies how the data is captured or generated. The citation statistics indicate academic impact; we report counts from both Google Scholar and Scopus, retrieved in June 2026. Availability is also reported: each dataset is classified as Public (directly downloadable), Request (accessible upon request), or Restricted (not found or unavailable). The access link or request page and the access date for every dataset are provided in Supplementary Table S1.

**Figure 5:**
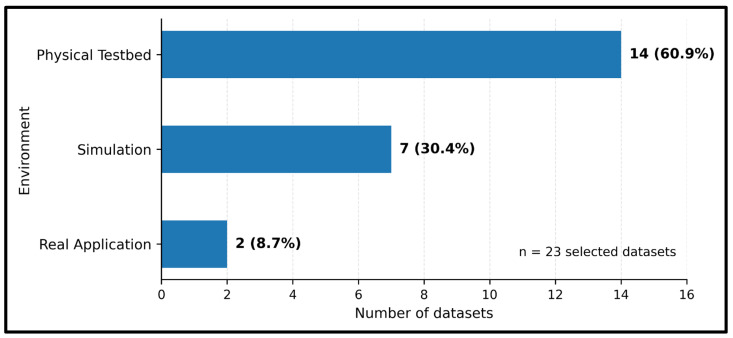
Environment distribution of studied datasets.

According to the chart in Figure 5, the majority of the OT datasets, 14 out of 23 (60.9%), were created with physical testbeds. This means that much of the comparative study is conducted with datasets produced in a controlled laboratory setting with real industrial parts; their realism is higher than that of purely synthetic data. The second group of datasets is simulation-based, comprising 7 datasets (30.4%), demonstrating that simulation is used to safely model attacks and complex industrial processes. Datasets collected from operational industrial infrastructures remain scarce owing to privacy, safety, and security constraints; Electra and ICS-NAD (2 datasets, 8.7%) are the only ones containing traffic from a real application. The implication for dataset selection is that the corpus is dominated by controlled-environment data: with only two datasets drawn from real operational environments, evaluations relying solely on testbed or simulated data may overstate detector performance relative to production deployments.

### 4.3. Dataset Generation Across the Selected Datasets

Across the 23 datasets, a few patterns stand out. Most are built on scaled-down water processes (treatment or distribution) or power and grid systems, with smaller numbers covering gas pipelines, chemical processes, manufacturing, railway traction, traffic control, and generic IIoT. Their controllers span the major OT vendors (Rockwell/Allen-Bradley, Schneider, Siemens, ABB, Emerson, and GE), alongside soft-PLC stacks (OpenPLC, ScadaBR) in the fully virtualized cases. Modbus is by far the most common protocol, with S7Comm, OPC UA, DNP3, EtherNet/IP, and IEC61850 appearing in a minority. In realism, the corpus ranges from real physical testbeds and hardware-in-the-loop rigs to pure process simulations and software-emulated SCADA sandboxes; only a few are drawn from real industrial applications, which is the main realism limitation discussed in Section 6. The per-testbed configurations are summarized in Table 4, and the individual datasets are described in the following subsections.

To keep the main text focused on the comparative analysis, the detailed per-dataset overview for all 23 datasets is provided in Supplementary Material S1 (Dataset Overview). The comparative characteristics of every dataset are presented across Table 3, Table 4, Table 5, Table 6, Table 7, Table 8 and Table 9.

## 5. Dataset Analysis

This section analyzes the 23 selected ICS-specific datasets along three axes: (i) the basic structural aspects (environment, feature type, format, and OT protocol coverage), (ii) the attack types reported and their mapping to MITRE ATT&CK for ICS tactics, and (iii) the feature dimensionality and class imbalance characteristics. The corresponding summary tables are presented sequentially below, with prose commentary highlighting patterns, gaps, and structural limitations across these datasets.

### 5.1. Basic Aspects: Environment, Features, Format, and OT Protocols

Table 5 summarizes four basic aspects of the 23 ICS-specific datasets: the context in which the data were generated, the types of features captured, the delivery format, and the OT protocols represented. The Environment column identifies data obtained from a physical testbed (a scaled-down version of an industrial process), data synthesized via simulation, and data from a live network in operation. The Features column indicates whether records contain network traffic (N), process (P), or both. The Format column indicates how each dataset is delivered (CSV tables, PCAP captures, or others).

The distribution of the environment type is heavily weighted toward testbeds, with 14 out of 23 datasets (60.9%) coming from testbeds (HAI is included here because its HIL augmentation overlays physical-testbed components), 7 from simulation (30.4%), and 2 datasets (8.7%) classified as Real Application: Electra and ICS-NAD. This dominance reflects the actual constraints that limit live-network capture in production ICS environments, and the persistent scarcity of operational data remains a fundamental obstacle to evaluating IDS realism in field conditions.

There are three categories of feature coverage: hybrid network-process (11 datasets, 47.8%), network-only (7 datasets, 30.4%), and process-only (5 datasets, 21.7%, including HAI). Of particular interest to IDS research is the hybrid set (SWaT, EPIC, S317, MSU-GP, MSU-PWR, ICS-Flow, Rodofile, Edge-IIoTset, HIL-WDT, IUNO, and EDS), which enables cross-layer correlation between network anomalies and process-state deviations. The most common delivery format is paired CSV/PCAP release (11 datasets, 47.8%), where raw captures are delivered alongside feature tables already prepared for ML. Other releases are pure CSV (8 datasets, 34.8%), usually process-only or feature-engineered. Less standard formats include R-Data (TEP) and undocumented formats (TLIGHT, IUNO, HiTar). Together, these account for only four datasets and raise reproducibility concerns where the underlying schema is not specified.

**Figure 6:**
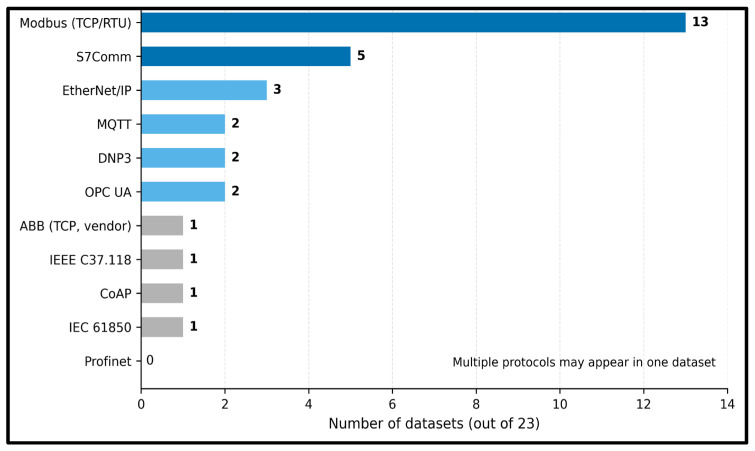
OT protocol coverage across the 23 selected datasets.

Protocol coverage is heavily concentrated. Modbus is found in 13 of 23 datasets, reflecting the widespread use of Modbus in industrial deployments. S7Comm is found in five datasets: Electra, Rodofile, ICS-NAD, TLIGHT, and EDS; EtherNet/IP is found in three datasets: SWaT, WADI, and S317; and DNP3 is found in two datasets: MSU-PWR and Rodofile. Two datasets (Edge-IIoTset, X-IIoTID) are relevant to IIoT scenarios where MQTT is mentioned. Several operationally important protocols are severely underrepresented: OPC UA is found only in IUNO and HAI, and Profinet does not appear in any selected dataset. Recent releases add some new coverage, with ICS-NAD adding three vendors (Modbus, S7Comm, and ABB over TCP), and EDS adding S7Comm-based industrial Ethernet traffic. However, the skew toward Modbus and away from OPC UA and Profinet protocols remains a structural limitation for transfer learning and protocol generalization studies. The frequency of each OT protocol across the datasets is shown in Figure 6.

Detectors trained mainly on Modbus traffic learn features that reflect Modbus semantics, such as function codes, register-address patterns, and the simple unauthenticated request and response structure of the protocol. These features, and the normal-traffic baselines they establish, do not carry over to protocols with different stacks and behaviors, for example, the job and data services of S7Comm, the service-oriented client and server sessions of OPC UA, or the GOOSE and MMS messaging of IEC 61850. A model whose decision boundary is tuned to Modbus is therefore unlikely to transfer without adaptation, and a detector that scores well on a Modbus benchmark may not retain that performance on a substation-automation or DCS deployment that uses other protocols.

The bias also shapes the attack surface that models learn: because Modbus is unauthenticated, false-data injection, command injection, and response manipulation dominate the available attack examples.

A parallel limitation applies to the process-data side of these datasets. Because each dataset is generated from one specific industrial process, the process features it exposes (sensor and actuator tags, their physical ranges, control set-points, and the steady-state dynamics of that plant) represent behavior that is particular to that process rather than to ICS in general. A detector trained on the water-treatment dynamics of SWaT therefore learns a normal-operation baseline that does not describe the gas-pipeline behavior of MSU-GP, the power-system measurements of MSU-PWR, or the chemical-process variables of TEP, even when the communication protocol is shared. Process-level generalization is thus constrained by process heterogeneity in the same way that network-level generalization is constrained by protocol heterogeneity, and the two effects compound when a target deployment differs from the benchmark in both process and protocol.

Consequently, single-protocol and single-process evaluation can overstate robustness, and generalization should be supported by multi-protocol and cross-process evaluation or by protocol-agnostic feature representations rather than inferred from Modbus-dominated, single-plant benchmarks.

### 5.2. Analysis of Attacks and Mapping to MITRE ATT&CK for ICS

The selected ICS datasets represent a range of attack scenarios. However, the scope, diversity, and level of detail of the reported attacks vary considerably across datasets. Therefore, examining the attack types included in each dataset is an important step in understanding their relevance for ICS intrusion-detection research.

In this study, the attacks reported in the 23 datasets were mapped to the MITRE ATT&CK for ICS tactic categories [18]. MITRE defines tactics as the adversary’s tactical objective, or the “why” behind an action, while techniques describe “how” that objective is achieved. Accordingly, the mapping in this study is based primarily on the apparent adversarial objective of each attack, rather than on the technical implementation alone. MITRE ATT&CK for ICS currently organizes ICS adversary behavior into 12 tactic categories: Initial Access, Execution, Persistence, Privilege Escalation, Evasion, Discovery, Lateral Movement, Collection, Command and Control, Inhibit Response Function, Impair Process Control, and Impact.

As shown in Table 6, the studied datasets cover a wide range of adversarial behaviors, from process-level manipulations (such as false data injection and actuator tampering) to network-level attacks (such as scanning, replay, man-in-the-middle, and DoS/DDoS). Most attacks target control and response functions, which reflect the cyber-physical nature of ICS security datasets. These tactics are especially relevant because many ICS attacks aim to manipulate control logic, disturb physical processes, mislead operators, or degrade system availability.

In contrast, datasets with broader IT/IIoT attack coverage, such as Edge-IIoTset, X-IIoTID, ICS-ADD, and HiTar, include attacks that extend beyond direct process manipulation. These datasets additionally cover tactics such as Initial Access, Execution, Persistence, Lateral Movement, Collection, and Command and Control. This indicates that the 23 datasets are not uniform in their attack representation: some are strongly process-centric, while others represent broader cyber or IIoT intrusion scenarios. Therefore, the choice of dataset should be aligned with the intended IDS research objective, whether the focus is on physical-process anomaly detection, network intrusion detection, or broader cyber-physical attack detection.

**Figure 7:**
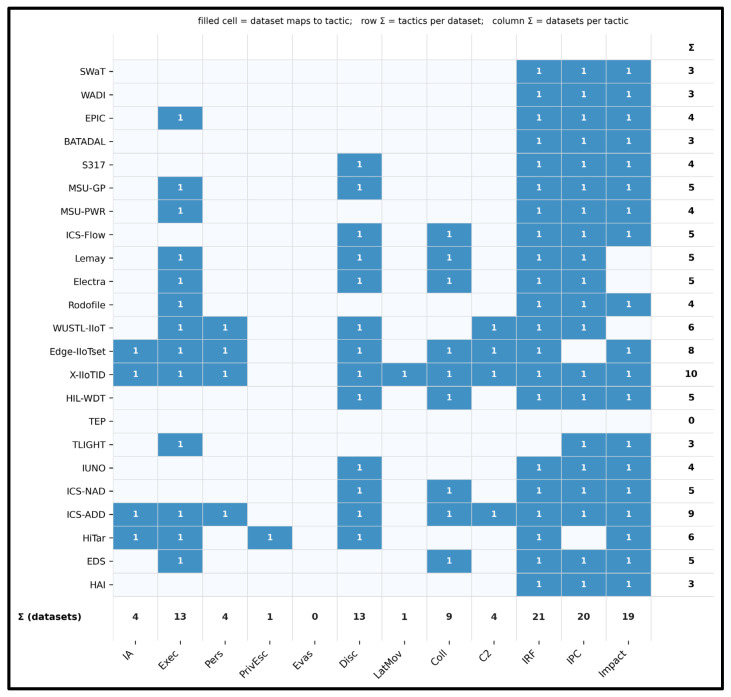
MITRE ATT&CK for ICS tactic coverage per dataset.

Three structural observations follow from Table 6. First, Discovery is the most consistently represented preparatory tactic across datasets that include any form of reconnaissance, scanning, or probing, including the entries for ICS-ADD and IUNO. Second, the Impact tactic is mapped in 19 of the 23 datasets, confirming that current ICS datasets predominantly capture attacks with observable operational consequences rather than purely stealthy or long-dwell behavior. Third, data-theft scenarios are underrepresented across the datasets and remain an open gap for future dataset releases. The per-dataset tactic coverage is visualized as a heatmap in Figure 7, with column sums highlighting Inhibit Response Function, Impair Process Control, and Impact as the most populated tactics, and Privilege Escalation, Evasion, and Lateral Movement as the structural gaps.

### 5.3. Feature Dimensionality and Class Imbalance

In machine learning-based intrusion detection, dataset characteristics such as feature dimensionality, sample size, and class distribution directly influence model performance. A high number of features may increase the model’s representational capacity, but it may also increase computational cost and the risk of overfitting if the dataset is not sufficiently large. Similarly, class imbalance is a common issue in ICS/OT intrusion-detection datasets, where normal operating data often dominate attack samples. This imbalance can bias learning algorithms toward the majority class and lead to misleadingly high accuracy while reducing the detection performance for minority attack classes. Previous studies have also highlighted that imbalance affects classifier performance and that metrics such as precision, recall, and F1 can be more informative than accuracy [58,59].

Table 7 summarizes the number of features, number of instances, anomaly percentage, and the normal-to-anomaly ratio for the selected datasets. Values marked as Not reported indicate that the corresponding information was not explicitly available in the dataset paper or public release documentation, and we could not derive it from the dataset files.

The results show that most datasets are imbalanced toward normal operation, which is expected in ICS environments where attacks are rare compared with stable process operation. However, some datasets, such as MSU-PWR (0.5:1) and X-IIoTID (1.06:1), show a more balanced distribution because they were generated with many attack scenarios. Therefore, model evaluation on these datasets should not rely only on accuracy. Instead, recall, F1-score, and macro-F1 should be reported to avoid overestimating model performance on majority classes.

Across Table 5, Table 6 and Table 7, three patterns deserve emphasis before turning to the documentation-completeness and decision-aid rubric analyses in Section 6. First, network-only datasets dominate the recent IIoT-oriented releases (ICS-ADD, HiTar, X-IIoTID), while hybrid network + process coverage remains concentrated in physical-testbed releases (EPIC, MSU-GP, MSU-PWR, HIL-WDT, EDS). Second, the most diverse attack inventories (Edge-IIoTset, X-IIoTID, ICS-ADD) belong to datasets explicitly designed around adversary lifecycles rather than process-specific scenarios, which have direct implications for benchmarking and APT-oriented (Advanced Persistent Threat) evaluation. Third, the imbalance picture splits the group into three classes: severely imbalanced ICS datasets (WADI, WUSTL-IIoT, Electra), moderately imbalanced datasets (SWaT, BATADAL, HIL-WDT, ICS-Flow), and near-balanced or attack-dominant datasets (MSU-PWR, Edge-IIoTset, X-IIoTID). The decision-aid scoring rubric in Section 6 makes these patterns explicit by inverse-scoring imbalance as a usability criterion, so that more balanced datasets score higher.

## 6. Results and Findings

The studied ICS datasets exhibit several limitations that can hinder IDS performance. These limitations fall into three categories: data representation, dataset management, and attack-scenario issues.

### 6.1. Data Representation Issues

Simulated and testbed environments may not realistically represent real-world ICS behavior, which is a limitation of such datasets. Models trained on them risk learning testbed-specific patterns rather than generalizable ones, which limits their transferability to operational deployments. A related concern is synthetically generated attack traffic, which may not reproduce the timing characteristics and protocol-level responses of live systems.

As shown in Table 8, most datasets are imbalanced toward normal operation, which complicates training, particularly for smaller datasets; the consequences for evaluation are discussed in Section 5.3. This imbalance is commonly mitigated through data-level methods such as oversampling and undersampling [60] and algorithm-level methods such as cost-sensitive learning.

A further representation gap is the absence of raw packet capture (PCAP) files in several datasets. Without raw data, researchers cannot recover low-level information such as inter-packet timing and protocol flags, which is often critical for protocol-aware detection methods.

### 6.2. Dataset Management Issues

A persistent challenge across these datasets is the absence of comprehensive documentation. Many datasets lack a system architecture description, a clear account of the attacks implemented, and details of the testbed configuration. Such information is essential for reproducibility and practical benchmarking.

Restricted availability compounds this problem. The sensitive nature of ICS environments leads some organizations to withhold detailed datasets to avoid exposing proprietary network configurations or operational procedures, limiting the data accessible to the broader research community. Availability is also unstable over time: some datasets were released publicly but can no longer be retrieved because their hosting repositories are no longer maintained, and for others, a request to the original authors went unanswered.

A further practical issue is dataset fragmentation, where several releases distribute traffic captures, labels, and metadata across multiple files. This increases the required effort for pre-processing and analysis.

### 6.3. Attack Landscape Issues

Attack realism is evaluated using the dataset descriptions and availability categories already reported in this manuscript. Based on Table 3, sixteen of the 23 selected ICS/IIoT-related datasets are publicly available, four are available on request, and three are restricted or not clearly obtainable. Most available labeled datasets contain attacks executed in controlled physical testbeds, hardware-in-the-loop environments, or simulations. This makes them valuable for IDS benchmarking, but it also means they should not be interpreted as uncontrolled real-incident datasets. Electra and ICS-NAD are the only two datasets classified in Table 3 as Real Application. Electra captures traffic from an electric traction substation in the railway sector, while ICS-NAD captures multi-vendor real-environment traffic with deliberately labeled attacks; neither is an uncontrolled incident capture.

The reviewed datasets also show uneven attack coverage. Traditional ICS datasets are mostly process-centric and emphasize false data injection, replay, actuator/sensor manipulation, command or response manipulation, DoS/DDoS, and reconnaissance. Newer IIoT-oriented datasets, such as Edge-IIoTset, X-IIoTID, ICS-ADD, and HiTar, extend the scope to encompass broader IT/OT intrusion behavior, including scanning, brute-force attacks, backdoors, command-and-control activity, ransomware-type activity, and privilege-oriented attacks. However, this broader coverage does not automatically imply stronger process realism; some of these datasets contain rich cyber labels but weaker closed-loop physical-process semantics.

Protocol dependence remains another limitation. Several attacks are tied to specific industrial communication stacks such as Modbus/TCP, S7Comm, EtherNet/IP, OPC UA, or IEC 61850. As discussed in the protocol analysis, Modbus-based datasets dominate the available datasets, while OPC UA, Profinet, and vendor-specific traffic are underrepresented. This skew limits the ability to claim protocol-level generalization from results obtained on a single dataset or protocol family.

Insider threats and multi-stage adversary behavior remain underrepresented. A few datasets provide richer adversarial structure, such as S317 through a live attack-defense exercise, X-IIoTID through lifecycle-oriented IIoT attack classes, and ICS-ADD through a Cyber Kill Chain-style sequence. Nevertheless, most datasets model short, isolated attack episodes rather than campaigns involving reconnaissance, initial access, persistence, lateral movement, process manipulation, operator deception, and physical impact. This constrains the evaluation of IDS methods aimed at detecting attacks that progress through several stages.

The documentation of attack generation is inconsistent. Some papers clearly describe the attack scenario and affected process variables, but do not always report the exact tools used. Capture, monitoring, parsing, and IDS/SIEM tools such as Wireshark, Zeek, Snort, OSSIM, Suricata, and OSSEC are treated as instrumentation rather than attack-generation tools unless the source explicitly identifies them as part of the attack execution. This distinction improves reproducibility and prevents overclaiming about dataset realism or adversary tooling [1,10].

The selected datasets support useful IDS benchmarking, but attack realism varies. Physical testbeds provide cyber-physical fidelity at the cost of scale; simulations offer safety and repeatability but lose authenticity; real-network captures remain scarce. When reporting IDS results, accuracy alone is misleading. Attack diversity, class imbalance, protocol coverage, and documentation quality should be reported alongside it.

### 6.4. Quantitative Analysis of Datasets Documentation

The documentation-completeness analysis evaluates how well each dataset can be understood, interpreted, and reused by other researchers. Following the 0–7 checklist defined in the methodology, one point is assigned for each documented element: system architecture, protocol/communication stack, attack methodology, record or class counts, feature description, labeling or ground-truth method, and availability conditions. The results show that documentation quality is generally strong across the selected datasets, with an average score of 6.04/7.

**Figure 8:**
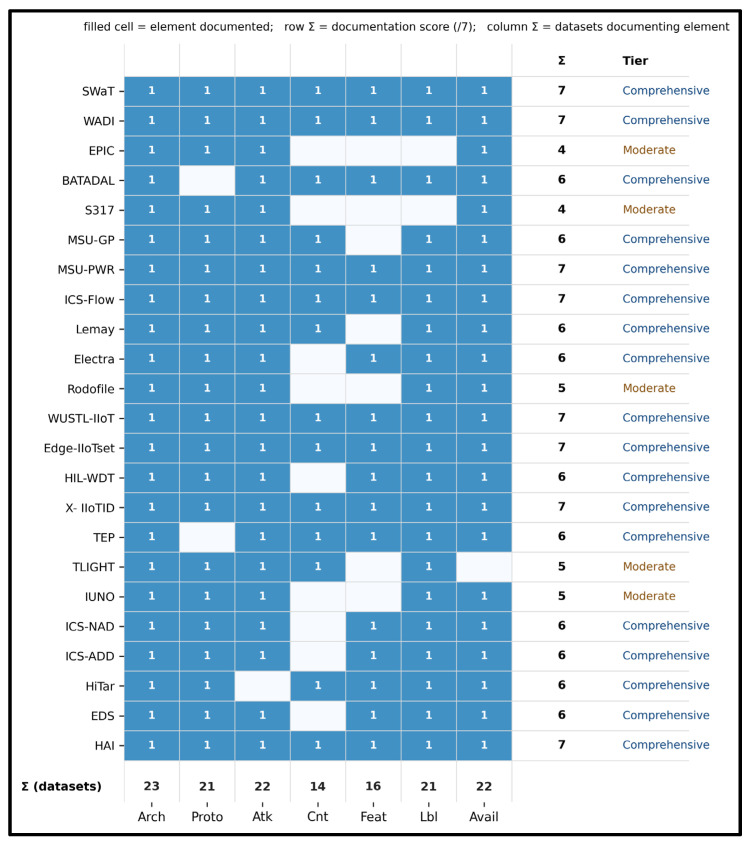
Documentation scoring heatmap. Documentation completeness is scored 0–7, one point per documented axis: architecture, protocol/communication stack, attack or anomaly methodology, counts, feature description, label/ground-truth method, and availability condition. Documentation tier cutoffs: 0–2 = Mild; 3–5 = Moderate; 6–7 = Comprehensive.

Figure 8 shows these per-dataset scores as a heatmap across the seven axes, and it also reveals recurring documentation gaps. The most common missing elements are detailed record/class counts and feature descriptions, while architecture, attack methodology, and availability are more frequently reported. This confirms that many ICS datasets are usable for benchmarking, but not always fully reproducible without additional preprocessing effort, manual interpretation, or consultation of repository files. This observation is consistent with prior ICS dataset surveys, which emphasize that dataset selection should consider not only realism and attack coverage, but also documentation, labeling transparency, availability, and reproducibility.

The seven documentation elements are weighted equally: each is a distinct, non-redundant, binary facet of dataset documentation, and there is no validated basis for ranking one above another, so equal weighting keeps the score transparent and reproducible while avoiding unvalidated subjective weights. However, we did not rely on this rationale alone: using a Monte Carlo weight-perturbation sensitivity analysis, we recomputed the documentation scores under 20,000 randomly sampled weightings, varying each element’s weight by up to ±50%, and the resulting documentation-completeness ranking remained almost identical to the equal-weight ordering (median Kendall’s τ = 0.91, Spearman’s ρ = 0.96). A reproducibility-oriented weighting that doubles the weight of availability, labeling, and record counts produced the same ordering (Kendall’s τ = 0.94). A one-at-a-time (leave-one-out) analysis, removing each element in turn, changed the ordering only marginally (Kendall’s τ between 0.76 and 1.00). The documentation ranking is therefore robust to the weighting scheme; tier labels for datasets at a category boundary are necessarily more sensitive, which is why the underlying 0 to 7 score is reported alongside the tier.

### 6.5. Decision-Aid Scoring Rubric and Per-Direction Recommendations

The taxonomic axes defined in Section 3 and applied in Table 3, Table 4, Table 7 and Table 8 describe each dataset from several perspectives. However, practitioners normally select a dataset for a specific task rather than for a generic comparison. This section converts the manuscript’s existing taxonomy into a transparent 0–15 decision aid. The rubric is not intended to identify a universal best dataset; it is intended to expose trade-offs among realism, attack diversity, imbalance usability, documentation, and reproducibility.

This interpretation is consistent with prior ICS dataset-survey guidance that dataset selection should consider the testbed or operational setting, protocol coverage, attack realism, documentation, availability, and suitability for the intended IDS task rather than relying on popularity or accuracy results alone [1]. It also reflects the imbalance concern discussed earlier in this manuscript, where precision, recall, F1-score, macro-F1, and PR-AUC are more informative than accuracy for skewed IDS datasets [58,59]. As an illustration, X-IIoTID achieves a near-balanced class distribution (1.06:1) that makes it directly convenient for supervised ML experiments, while datasets such as WADI or WUSTL-IIoT exhibit severe imbalance that requires explicit sampling or class-weighting strategies.

#### 6.5.1. Rubric Definition

Each dataset is scored from 0 to 3 on five criteria. The total composite score is the sum of the five criteria, with a nominal maximum of 15. For datasets where the Imbalance ratio is neither stated in primary documentation nor extractable from the released dataset files, the Imbalance Usability axis is excluded from the composite, and the dataset’s total is renormalized to /12 (also reported as a percentage of the achievable maximum). Equal weighting is used to keep the rubric auditable; the final recommendation still depends on the research direction.

- Realism: derived from the Realism column in Table 8, which is based on the dataset descriptions and the environment/evidence reported in Table 3. The score jointly reflects the operational realism of the environment and the provenance of the attack traffic. Scoring: 3 = real operational network with naturally occurring or incident-captured attack traffic that was not deliberately introduced by the dataset authors; 2 = real operational environment in which the attack traffic was deliberately introduced by the dataset authors; 1 = deliberately injected attacks in a controlled physical or hardware-in-the-loop testbed rather than an operational network; 0 = synthetic or pure-simulation environment with no physical hardware in the loop. Because Realism = 3 requires naturally occurring or incident-captured traffic, no dataset reaches the nominal maximum composite (15/15 for datasets with reported Imbalance, 12/12 for datasets where Imbalance is excluded). Edge-IIoTset’s 86.7% is the highest score achieved.
- Attack diversity: derived from the Diversity column in Table 8. Scoring: 3 = Diverse (five or more tactics); 2 = Moderate (three to four tactics); 1 = Mild (one to two tactics); 0 = not applicable or not cyber-attack-oriented, as in pure process-fault datasets.
- Imbalance usability: derived from the Imbalance column in Table 8 but inverse-scored. Scoring: 3 = Mild (normal-to-attack ratio ≤ 4:1); 2 = Moderate (4:1 to 9:1); 1 = Severe (>10:1); NR (not reported) = this axis is excluded from the composite for that dataset, and the total is renormalized to /12 (see introductory paragraph). These cut-offs align with the thresholds applied in Table 8.
- Documentation: derived from the documentation-completeness score in Figure 8. Scoring: 3 = 7/7; 2 = 5–6/7; 1 = 3–4/7; 0 = 0–2/7.
- Reproducibility: derived from the Availability column in Table 3 and the Format column in Table 5. Scoring: 3 = public dataset with raw artifacts available (PCAP for network-oriented datasets, raw time-series for process-oriented datasets, or both); 2 = public feature-only or preprocessed release, or request-based access with raw artifacts; 1 = request-based access feature-only; 0 = restricted, offline, or not clearly obtainable.

Table 9 presents the rubric scores, sorted by percentage of achievable maximum in descending order. Where multiple datasets share the same percentage, the secondary sort is by Documentation (higher first), then Realism, then Reproducibility, then the original serial number.

**Figure 9:**
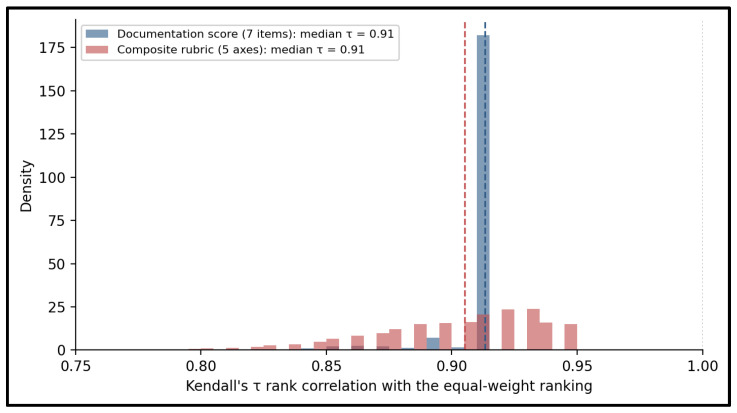
Ranking stability under random reweighting. Dashed vertical lines mark the median Kendall’s τ of the distribution.

Sensitivity and robustness of the composite scores. The five criteria are combined as an unweighted sum, again to keep the rubric auditable and free of unvalidated subjective weights, with no criterion privileged a priori. To verify that the ranking does not depend on this choice, we performed a Monte Carlo weight-perturbation sensitivity analysis. Recomputing the percentage-of-achievable ranking over 20,000 randomly sampled weightings, with each axis weight varied by up to ±50%, left the composite rubric ranking highly stable (median Kendall’s τ = 0.91), as shown in Figure 9, with the leading group (Edge-IIoTset, ICS-NAD, and X-IIoTID) retained as the top three in 95% of the draws; the residual exchange of the first two positions reflects the exact tie between Edge-IIoTset and ICS-NAD (both 13/15) rather than instability. Emphasizing any single criterion by 25% leaves the leading datasets unmoved and shifts only mid-ranked datasets by one or two positions (for example, reproducibility emphasis lifts TEP, and attack-diversity emphasis lifts HIL-WDT, MSU-GP, and EDS). No single criterion dominates: a one-at-a-time (leave-one-out) analysis, removing each axis in turn, left the ranking strongly correlated with the full rubric (Kendall’s τ between 0.74 and 0.90, lowest when Reproducibility is removed). Under a more aggressive test that perturbs the discrete axis scores by one band for a quarter of the entries (emulating threshold and judgment uncertainty), the ranking remained strongly and positively correlated with the original (median Kendall’s τ = 0.78) and the broad tiers were preserved, while only the fine positions among closely scored datasets shifted. These results justify the equal-weighting choice and reinforce the guidance in this section that the composite total is an indicative decision aid rather than an absolute ordering. As Figure 9 shows, the rank-correlation values concentrate near the upper end (median Kendall’s τ = 0.91), with few perturbations producing a materially different order. The practical implication is that a user of the decision aid can rely on the published ranking without tuning the axis weights: the leading datasets stay the leaders across almost all weightings, so the recommendation is robust to reasonable disagreement about the relative importance of the five criteria.

#### 6.5.2. Per-Use-Case Recommendations

The recommendations below interpret the rubric in terms of five common research and engineering tasks. A dataset is recommended when it meets the primary criteria for that task; where no dataset satisfies all criteria, the limitation is stated explicitly.

(1) Training and general supervised IDS development: Priority criteria: documentation and reproducibility. The strongest choices are Edge-IIoTset [39], X-IIoTID [41], MSU-PWR [29,30], SWaT [19], MSU-GP [27,28], ICS-Flow [11], and BATADAL [25]. These datasets combine usable documentation with accessible or requestable artifacts and a non-severe class distribution. WUSTL-IIoT [36] and WADI [22] remain useful, but their severe imbalance requires careful sampling, class weighting, or metric selection. TEP [42] is suitable for process-anomaly or fault-detection studies, but it should not be presented as a full cyber-network IDS benchmark.
(2) Benchmarking and comparative evaluation across publications: Priority criteria: realism, documentation, and attack diversity. ICS-NAD [46] is the strongest candidate, scoring 3/3 on attack diversity, imbalance usability, and reproducibility and 2/3 on realism and documentation (13/15 = 86.7%; see Section 6.5.1). Realism is capped at 2/3 because no dataset in this corpus contains naturally occurring incident traffic, and ICS-NAD’s class balance is reported only for its Siemens subset. Electra [33] is also valuable for realistic railway traction-substation traffic with diverse attacks, but its restricted availability reduces reproducibility. Because very few datasets meet all three benchmarking criteria, a practical benchmark suite should pair realism-focused datasets such as ICS-NAD or Electra with high-reproducibility datasets such as Edge-IIoTset [39], X-IIoTID [41], HIL-WDT [40], ICS-Flow [11], or ICS-ADD [47].
(3) Protocol-specific studies: Priority criteria: the target protocol, documentation, and reproducibility. For Modbus: ICS-Flow [11], HIL-WDT [40], ICS-NAD [46], ICS-ADD [47], Edge-IIoTset [39], X-IIoTID [41], Lemay [32], MSU-GP [27,28], WUSTL-IIoT [36]. For S7Comm, the strongest accessible choices are Rodofile [34], ICS-NAD [46], and EDS [49], with TLIGHT [43] as a specification-level reference for Siemens S7 PLC scenarios. For EtherNet/IP, SWaT [19], WADI [22], and S317 [26] are the relevant iTrust-linked candidates, subject to request access and artifact availability. For IEC 61850, EPIC [24] is the only selected candidate, but its documentation and the limitations of the released schema should be acknowledged. For OPC UA, IUNO [44] and HAI [50,51] are the most relevant candidates, although availability and file-structure limitations reduce reproducibility. For DNP3, MSU-PWR [29,30] and Rodofile [34] are the relevant selected datasets. For MQTT/CoAP-oriented IIoT studies, X-IIoTID [41] and Edge-IIoTset [39] are the main candidates.
(4) IIoT and smart-manufacturing contexts: Priority criteria: IIoT-relevant architecture or protocols, attack diversity, and documentation. X-IIoTID [41] and Edge-IIoTset [39] are the choices because they were designed around heterogeneous IIoT environments and contain broad attack taxonomies. WUSTL-IIoT [36] is appropriate for water-storage/Modbus-based IIoT intrusion detection with clear documentation, but imbalance must be handled carefully. ICS-ADD [47] is useful when the study requires SCADA/PLC traffic together with SIEM/NIDS-style monitoring outputs. HiTar [48] is suitable for log-based smart-manufacturing or transfer-learning studies.
(5) APT, multi-stage, and kill-chain-oriented evaluation: Priority criteria: attack diversity and realism, with special attention to whether the dataset captures staged adversary progression rather than isolated attack episodes. No selected dataset fully represents real APT campaigns in operational ICS networks. ICS-NAD [46] is the best realism/diversity candidate, and S317 [26] contributes a live attacker-team exercise context. X-IIoTID [41] and ICS-ADD [47] are recommended as structured alternatives because they include lifecycle or Cyber Kill Chain-style attack organization, even though their attacks are deliberately injected. Electra [33] can support this use case when realistic substation traffic is required and access is available. Edge-IIoTset [39] adds a broad attack variety, but it should be used cautiously for APT claims because the breadth of attack labels is not the same as a validated multi-stage ICS campaign. HAI [50,51] complements these candidates for studies focused on stealthy process-level attacks rather than network-protocol exploits, particularly from the HAI 22.04 release onward.

**Figure 10:**
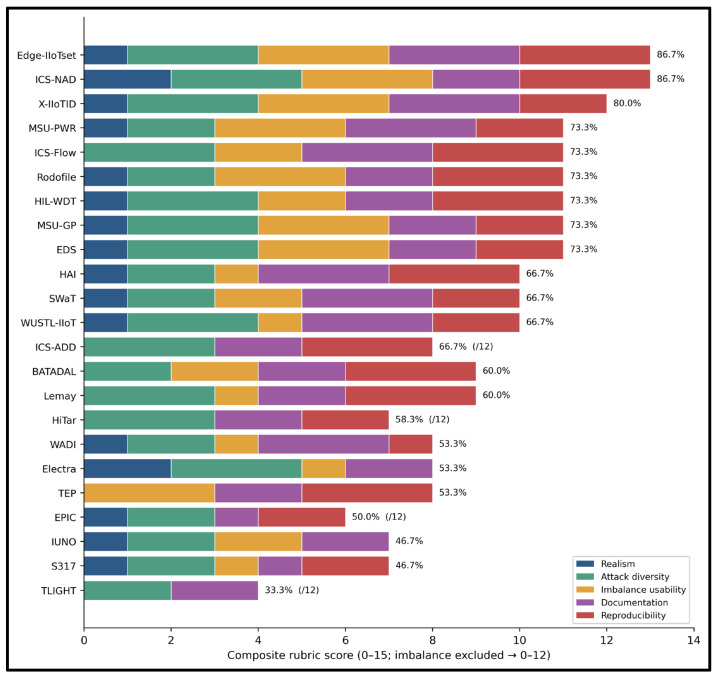
Use-case rubric composite scores per dataset.

**Figure 11:**
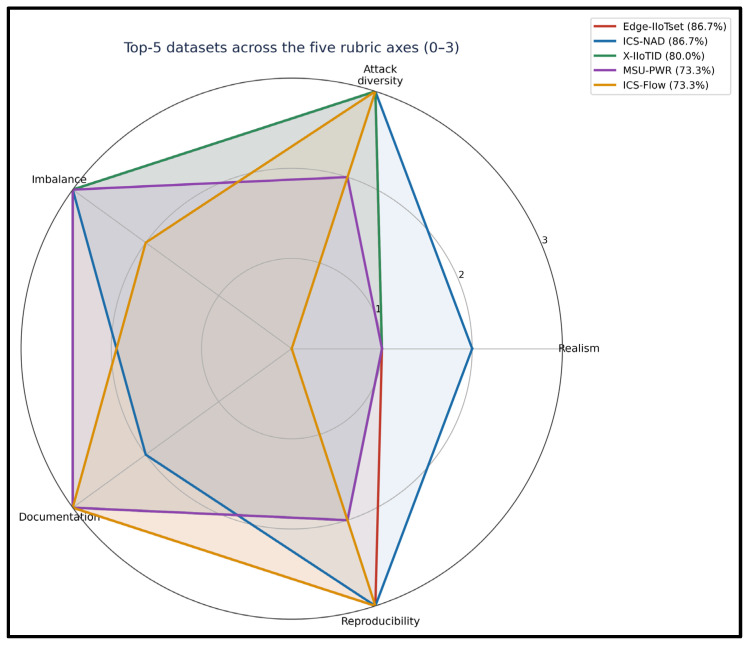
Radar comparison of the five top-ranked datasets across the five rubric axes.

Overall, Edge-IIoTset and ICS-NAD lead the composite ranking at 86.7% (13/15), and X-IIoTID follows at 80% (12/15), with the remaining datasets spanning 73.3% down to TLIGHT at 33.3%. Figure 10 presents the full ranking, with each bar segmented into the five rubric criteria. Figure 11 complements this ranking by comparing the five highest-scoring datasets across the five rubric axes. The profiles show that these datasets reach comparable totals through different strengths rather than by leading on every axis: all five score highly on attack diversity, imbalance usability, and documentation, but the visible dents fall almost entirely on the realism axis (none exceeds 2, and ICS-Flow scores 0), reflecting the absence of naturally occurring attack traffic noted in Section 6.5.1. The leaders, therefore, trade strengths; for example, ICS-NAD contributes the group’s only operational realism while giving up some documentation, whereas Edge-IIoTset and X-IIoTID maximize documentation, which is why the recommendations below pair a realism-anchored release with a documentation- or reproducibility-strong one rather than relying on any single benchmark.

However, the total score should never be used alone. A lower-scoring dataset can still be the most appropriate choice when it uniquely matches the required domain or protocol; for example, EPIC remains important for IEC 61850-oriented smart-grid studies, IUNO for OPC UA-focused studies, and TEP for process-fault/anomaly detection. Conversely, a high total score does not guarantee process realism or suitability for every OT deployment. This avoids overclaiming generalization from narrow, restricted, or weakly documented datasets while still allowing specialized datasets to be used where they provide unique value.

### 6.6. Hierarchical Dataset Categorization

**Figure 12:**
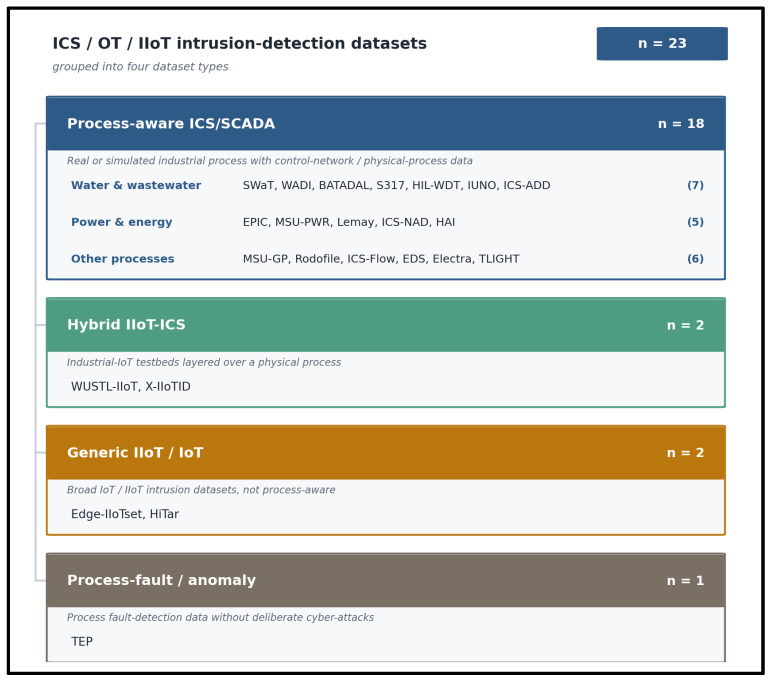
The 23 datasets are grouped into four types (the process-aware type is split by sector). Badge values (n) give the dataset count per type, and the total (n = 23); the parenthetical values (7, 5, 6) are the counts for the water-and-wastewater, power-and-energy, and other-process sectors.

The 23 datasets are not equally comparable: some capture a physical industrial process, while others are generic IoT traffic. To make this distinction explicit, we organize the corpus into a four-way hierarchy (Figure 12, Table 10): process-aware ICS/SCADA datasets, further grouped by sector into water and wastewater, power and energy, and other processes; hybrid IIoT–ICS datasets; generic IIoT/IoT datasets; and process-fault/anomaly datasets.

This separation matters for interpretation. In particular, Edge-IIoTset and HiTar are generic IIoT/IoT datasets and are not process-aware, so their attacks and features are not directly comparable to the process-level evaluation supported by ICS/SCADA datasets such as SWaT or HAI; a detector that performs well on Edge-IIoTset should not be assumed to transfer to a process-aware ICS setting without re-evaluation. Likewise, TEP is a process-fault benchmark without deliberate cyber-attacks, and the hybrid IIoT–ICS datasets sit between these extremes. The comparisons and rankings in this review should therefore be read primarily within, rather than across, these categories wherever process fidelity is the deciding factor.

### 6.7. Quantitative Cross-Dataset Analysis

**Figure 13:**
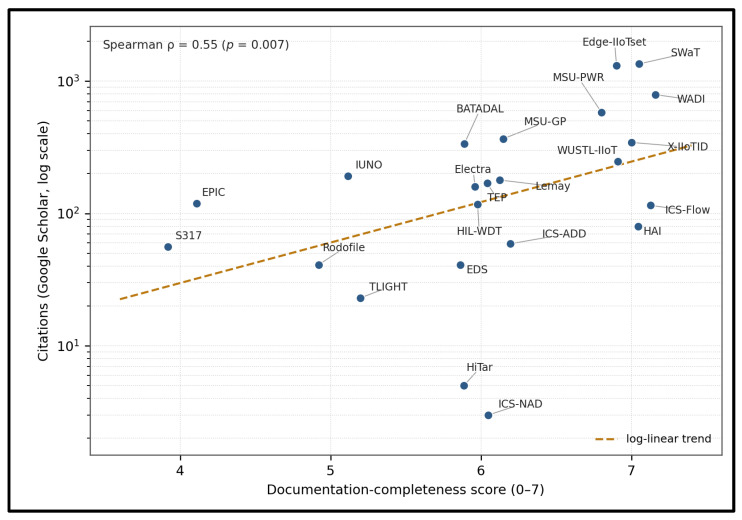
Documentation score vs. citation count for the 23 datasets.

To complement the qualitative comparison, we performed quantitative analyses using only the values already tabulated in this review. The first tests whether better-documented datasets are also the ones the community reuses, by relating each dataset’s documentation-completeness score (Figure 8) to the citation count of its primary reference (Table 3). We quantify the relationship with two correlation coefficients, each ranging from −1 (perfect inverse) to +1 (perfect agreement), where 0 means no association: Spearman’s ρ, which measures whether the two quantities rise together in rank order and is robust to the heavily skewed citation counts, and Pearson’s *r*, which measures a straight-line association on the values themselves. Both indicate a moderate and statistically significant positive relationship (Spearman ρ = 0.55, *p* = 0.007; Pearson *r* = 0.51), and the same holds against the Scopus counts (ρ = 0.55, *n* = 21); the close agreement between the two coefficients indicates the trend is not driven by a few highly cited datasets. This association is not an artifact of dataset age, since the documentation score is uncorrelated with publication year (*r* = 0.17, not significant) even though newer datasets have naturally accrued fewer citations. More completely documented datasets, therefore, tend to be reused and cited more, although we report this as an association rather than a causal claim (Figure 13).

**Figure 14:**
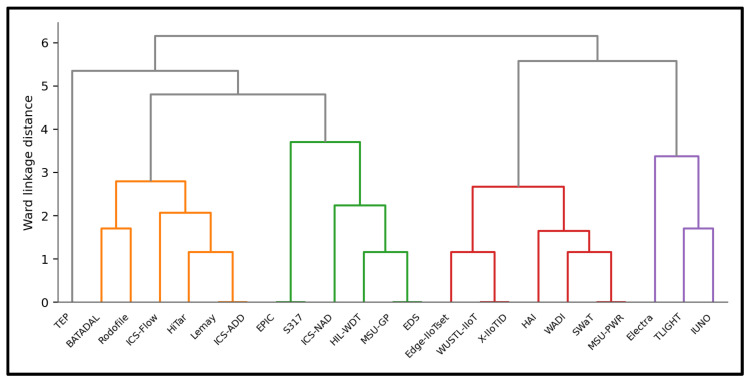
Hierarchical clustering of the 23 datasets on the standardized rubric dimensions. Branch colors denote the clusters formed below the linkage-distance cut; grey links join those clusters at higher distances.

**Figure 15:**
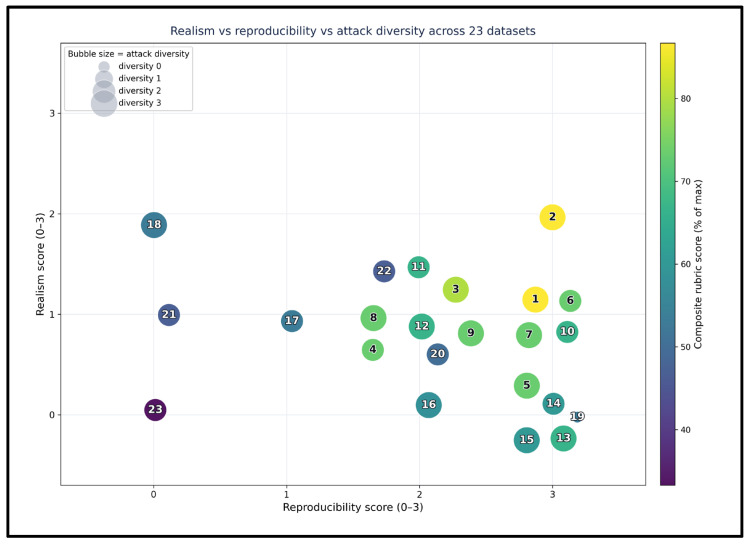
Realism versus reproducibility; bubble size encodes attack diversity; color encodes the composite rubric score. Datasets are numbered by composite rank: 1, Edge-IIoTset; 2, ICS-NAD; 3, X-IIoTID; 4, MSU-PWR; 5, ICS-Flow; 6, Rodofile; 7, HIL-WDT; 8, MSU-GP; 9, EDS; 10, HAI; 11, SWaT; 12, WUSTL-IIoT; 13, ICS-ADD; 14, BATADAL; 15, Lemay; 16, HiTar; 17, WADI; 18, Electra; 19, TEP; 20, EPIC; 21, IUNO; 22, S317; 23, TLIGHT.

Because the rubric in Section 6.5 is an authored instrument with equally weighted axes, we add an unsupervised cross-check that uses none of those weights: hierarchical clustering lets the datasets group by their own axis values, so any recovered structure reflects the data rather than our scoring choices. We grouped the datasets by their taxonomy dimensions using Ward linkage on the standardized realism, attack-diversity, documentation, and reproducibility axes; the imbalance-usability axis was excluded because it is not reported for four datasets. Three groups emerge (Figure 14): a comprehensively documented benchmark group (SWaT, WADI, MSU-PWR, WUSTL-IIoT, Edge-IIoTset, X-IIoTID, and HAI); a larger group of reproducible simulation and testbed datasets with thinner documentation (EPIC, BATADAL, S317, MSU-GP, ICS-Flow, Lemay, Rodofile, HIL-WDT, TEP, ICS-NAD, ICS-ADD, HiTar, and EDS); and a small group of limited-availability datasets (Electra, TLIGHT, and IUNO). That this assumption-light method recovers the same documentation and availability split confirms the finding is a property of the corpus, not an artifact of the rubric, and supports treating documentation completeness and availability as the primary differentiators between datasets. In addition, Figure 15 plots realism against reproducibility for the 23 datasets, with bubble size encoding attack diversity, and shows that high realism and high reproducibility rarely coincide in the same release.

To probe the rubric axes further, we examined two additional relationships using the tabulated scores. First, because two axes that always move together would be partly redundant, we tested whether documentation and reproducibility capture the same underlying property. Across the corpus, they are essentially uncorrelated (Spearman ρ = 0.05, *p* = 0.83): a dataset can be thoroughly documented yet hard to reproduce, or reproducible yet sparsely documented. This supports treating them as separate criteria. Second, to confirm that the composite ranking reflects dataset quality rather than dataset type, we compared the composite scores across the four dataset categories of Section 6.6. The difference is not statistically significant (Kruskal–Wallis *H* = 2.41, *p* = 0.49; process-aware versus other datasets, Mann–Whitney *p* = 0.47), so dataset quality as captured by the rubric does not differ systematically by category, although the small and uneven category sizes (18, 2, 2, and 1) limit the power of the test. Finally, the descriptive growth in dataset releases over time is shown in Figure 4; because the corpus is cross-sectional and small, with only two datasets from real operational environments, we do not infer a temporal evolution of dataset properties.

## 7. Discussion and Future Directions

The analysis in Section 4, Section 5 and Section 6 highlights both the contributions and the structural limitations of the current ICS/IIoT datasets. This section first synthesizes the emerging themes and trends from the review (Section 7.1), then sets out future research directions (Section 7.2), organized around six themes and concludes with the study’s limitations (Section 7.3). The six themes are: (i) standardization and reporting, (ii) hybrid and ensemble learning architectures, (iii) federated learning and privacy-preserving collaboration, (iv) cross-domain transfer learning, (v) explainable AI for operator-facing IDS, and (vi) emerging data and learning paradigms (graph-based detection, streaming and online learning, LLM-assisted reasoning, foundation models, digital twins, synthetic data generation, and quantum intrusion detection).

### 7.1. Emerging Themes and Research Trends

Several themes characterize the current ICS/IIoT dataset landscape and the trends shaping its development. First, protocol coverage is concentrated: Modbus dominates the corpus, while protocols that are widespread in industry, such as S7Comm, OPC UA, and IEC 61850, remain comparatively underrepresented, which biases benchmarks toward Modbus-specific behavior. Second, realism is largely testbed-bound: most datasets are produced on physical or hardware-in-the-loop testbeds and only two capture real operational environments, so high realism and high reproducibility rarely coexist in one release and genuine operational-incident traffic is scarce. Third, adversary coverage is uneven: although the attacks of almost every dataset map to MITRE ATT&CK for ICS (22 of 23), coverage concentrates on the impact-oriented tactics (Inhibit Response Function, Impair Process Control, and Impact), while multi-stage and early-kill-chain behavior is sparsely represented. Fourth, documentation quality is improving but uneven, and it tracks scholarly uptake: documentation completeness correlates with citation count, and the best-documented, machine learning-ready releases, such as Edge-IIoTset, are also among the most adopted. Finally, the dataset population is diversifying beyond classical SCADA testbeds toward hybrid IIoT-ICS, edge and cloud, and process-fault datasets, with recent releases increasingly packaging artifacts (raw captures, feature schemas, and security-tool outputs) that support emerging methods such as graph-based, federated, and LLM-assisted detection. Together, these themes indicate that future dataset development will likely prioritize protocol and operational diversity, richer multi-stage attack scenarios, and standardized, well-documented, ML-ready releases.

### 7.2. Future Directions

Standardization is the most obvious gap. Across the 23 selected datasets, attack labeling, feature schemas, and protocol coverage are inconsistent, and the documentation-completeness scoring presented in Section 6.4 shows that even datasets with strong adoption omit individual reporting axes. Cross-dataset comparison would benefit from a community-agreed minimal reporting standard. Aligning future dataset releases with established cybersecurity taxonomies such as MITRE ATT&CK for ICS [18] and the IEC 62443 [61] control reference would also improve attack labeling and enable consistent cross-corpus mapping; this alignment is a future-directions item rather than a contribution of the present manuscript.

Hybrid and ensemble learning architectures, in which two or more model families address different signal regimes, are a promising direction for ICS-IDS. Stacked combinations of supervised classifiers with anomaly-based detectors can simultaneously cover the well-labeled attack space and the long-tail unknown space, which is poorly represented in current datasets [15,16].

Federated learning is an important direction for ICS environments where data sharing is constrained by safety, regulation, or competitive concerns. Federated approaches allow multiple plants or operators to jointly train detection models without exchanging raw traffic. The corpus surveyed in this manuscript already contains datasets explicitly designed to support federated evaluation (Edge-IIoTset [39]), which provides a starting point. Open questions include client-drift handling for non-IID OT process distributions, robustness to malicious participants, and the evaluation of communication-efficient aggregation strategies on realistic ICS feature dimensionalities.

Cross-domain transfer learning addresses a different but related problem: the scarcity of labeled attack traffic in any single ICS environment. Models trained on one dataset rarely transfer cleanly to another because of differences in process dynamics, protocol mix, and adversary tooling. For example, a model trained on the Modbus-based datasets in this review, such as ICS-Flow, HIL-WDT, or MSU-GP, would need domain adaptation before it could be applied to the S7Comm data of Rodofile and EDS or the IEC 61850 traffic of EPIC. These dataset pairs are therefore good candidates for studying cross-domain and cross-protocol transfer. The skew toward Modbus identified in Section 5.1 and the absence of operational-incident captures identified in Section 6.3 reinforce this challenge. Within this corpus, IEC 61850-specific cross-domain transfer learning is particularly underexplored; EPIC [24] is the only IEC 61850-bearing dataset in the selected set, and the broader literature on IEC 61850-to-IEC 61850 or IEC 61850-to-Modbus transfer remains thin. This is an attractive direction for future work because IEC 61850 deployments are operationally important (substation automation, smart-grid protection) but underrepresented in benchmarks. Beyond protocols, the same corpus supports cross-process transfer studies: pairs such as SWaT or WADI (water treatment and distribution) against MSU-GP (gas pipeline), MSU-PWR (power system), or TEP (chemical process) differ primarily in process physics rather than protocol, and are natural testbeds for learning process-invariant representations or domain-adaptation methods that carry a detector from one industrial process to another.

Explainable AI for IDS is a further key research direction worth highlighting. Operator trust and incident-response actions depend on the IDS providing not only a binary alarm, but also an account of the input features, protocol fields, or process variables that drove the alarm. Datasets that release both raw artifacts and feature schemas (Edge-IIoTset [39], ICS-NAD [46], HIL-WDT [40], ICS-Flow [11]) are best suited for XAI evaluation because they allow attributions to be linked back to interpretable OT signals. Reducing false-alarm rates through XAI-informed feature pruning and operator-feedback loops is closely tied to the imbalance and documentation challenges discussed earlier in this manuscript.

In addition to the directions above, which already include federated learning and explainable AI, several emerging learning paradigms place new demands on dataset design that the current corpus only partly meets. First, graph-based intrusion detection with graph neural networks requires datasets that expose the communication or process topology explicitly, for example, device and flow graphs in which nodes are PLCs, sensors, or hosts and edges are their interactions; the reviewed datasets are delivered mainly as flow tables or process time-series and rarely release this structure. Second, online and streaming detection, which reflects how an IDS actually operates in production under concept drift, needs ordered, timestamped streams with drift annotations; most reviewed datasets are static, pre-partitioned captures, so although several provide timestamps, few are framed for continual or online-learning evaluation. Third, LLM-assisted cyber reasoning, in which language models help interpret and triage alerts, needs telemetry paired with textual security context such as alert logs, security-tool outputs, or analyst annotations; ICS-ADD, which releases SIEM and NIDS outputs alongside raw traffic, is an early step in this direction, but such paired textual context is otherwise scarce. Releasing datasets that carry graph structure, drift-aware ordering, and aligned textual context would make these emerging directions tractable for ICS intrusion detection.

Beyond these, three further directions also depend on the data available for training and evaluation. Foundation models pre-trained on large, multi-site operational and protocol datasets and then adapted to a target plant could lower the per-deployment labeling burden, but they require broad cross-site data that the current corpus does not provide. Digital twins, high-fidelity virtual replicas of a physical process, offer a safe route to generate labeled attack data at scale, including the operational-incident captures that are scarce today. Relatedly, synthetic data generation with generative adversarial or diffusion models can rebalance rare attack classes and augment small datasets, provided the generated traffic is validated against real process behavior.

A further interesting direction is quantum intrusion detection (QIDS), which applies quantum machine learning and quantum-enhanced anomaly detection to pattern recognition and large-scale threat analysis [62]. Its progress is currently constrained by the dataset foundations emphasized in this review: existing QIDS studies rely on simulated quantum back-ends and on legacy or classical IDS datasets, and no dedicated quantum-cybersecurity dataset yet exists for ICS or OT [63].

### 7.3. Limitations

Beyond the dataset-level issues identified in Section 6, several limitations of this review itself should be noted. First, the scoring instruments involve a degree of judgment: although the documentation-completeness checklist and the decision-aid rubric use explicit criteria, and the attack-diversity axis is tied to the MITRE ATT&CK for ICS tactic count, the assignment of realism levels and of the documentation axes still requires interpretation of each source, so independent reviewers might differ on borderline cases. The sensitivity analysis in Section 6.5.1 shows that this judgment does not materially change the ranking, and structured expert validation of the rubric criteria and weights remains a useful direction for future work.

Second, dataset availability evolves over time: links are deprecated, access conditions change, and new releases appear, so the availability classifications and the corpus itself represent a snapshot (datasets accessed in May 2026) rather than a permanent state. Third, the review necessarily covers datasets that are publicly available or available on request; proprietary and operational datasets held by industry are not accessible to us and are therefore underrepresented, which biases the analysis toward shareable, mostly testbed-based data and away from real operational-incident traffic. Fourth, this is a narrative taxonomic review rather than a systematic PRISMA review, and although the selection followed explicit inclusion and exclusion criteria, it was performed by the authors and may not capture every relevant dataset.

Finally, several quantitative fields are reported as Not Reported because the originating publications do not disclose them, and the modest number of datasets, with only two having real operational provenance, limits the statistical analyses that can be reliably supported. We retain reported gaps explicitly rather than estimate values.

## 8. Conclusions

This study presented a taxonomic review of 23 ICS/OT/IIoT-related datasets used in machine learning-based intrusion detection research. The datasets were analyzed along seven measurable axes (environment, data type, format, OT protocols, attack representation, feature/class structure, and documentation), and their reported attacks were mapped to MITRE ATT&CK for ICS tactics. Two quantitative instruments were introduced: a 0–7 documentation-completeness checklist (Section 6.4) and a 0–15 decision-aid scoring rubric covering realism, attack diversity, imbalance, documentation, and reproducibility (Section 6.5). Both are fully derived from the public release documentation of each dataset and the analytical tables in this manuscript.

Several structural findings stand out, which Section 7.1 synthesizes as emerging themes: realism is concentrated in testbeds, with only two datasets drawn from real operational environments; OT protocol coverage is skewed toward Modbus; multi-stage and APT-style adversary behavior is underrepresented; and documentation, though generally strong, is uneven. One feature warrant separate emphasis here because it directly shapes evaluation: class imbalance is the dominant statistical characteristic of ICS datasets, with consequences for the choice of evaluation metric and for the comparability of reported IDS performance.

Based on these findings, no single dataset achieves the maximum score on all rubric criteria. Edge-IIoTset [39] (86.7%, 13/15) emerges as the strongest general-purpose IIoT release, followed by ICS-NAD [46] (86.7%, 13/15), the strongest realism-anchored candidate, and X-IIoTID [41] (80.0%, 12/15). HAI [50,51] (66.7%, 10/15) remains the strongest stealthy process-attack release. For practical benchmarking, the recommended approach is to pair a realism-focused release (ICS-NAD, Electra) with a documentation- or reproducibility-strong release (Edge-IIoTset, X-IIoTID, HIL-WDT, ICS-Flow, ICS-ADD) and to report results against both rather than a single benchmark.

The most pressing open challenges for the ICS dataset community are: (i) generating or releasing operational-incident traffic from production networks, within the legal and safety-disclosure constraints that govern such releases, (ii) broadening OT protocol coverage to include OPC UA, IEC 61850, and Profinet at usable volume, (iii) capturing multi-stage adversary campaigns with kill-chain-aware labels, and (iv) adopting a community-agreed minimal reporting standard that aligns labels with MITRE ATT&CK for ICS and with IEC 62443 reference models. The future research directions identified in Section 7 (federated learning across plants, cross-domain transfer learning, especially for IEC 61850, and explainable AI for operator-facing IDS) depend critically on the availability of datasets that satisfy these standardization and realism requirements.

## Figures and Tables

**Table 1:** Comparison with recent dataset surveys.

Survey (Year)	Primary Focus	Datasets Focus	Coverage	Doc. Score	Use-Case Rubric	ATT&CK-ICS Map	Use-Case Ranking
Choi et al. [8] (2019)	Comparison of ICS datasets by attack paths	Primary	Till 2017	✗	✗	✗	✗
Conti et al. [1] (2021)	ICS testbeds and datasets survey	Primary	Till 2020	✗	✗	✗	✗
Koay et al. [5] (2023)	Survey of ML for ICS security	Secondary	Till 2020	✗	✗	✗	✗
Mitseva et al. [10] (2023)	Representativeness of ICS datasets	Primary	Till 2022	✗	✗	✗	✗
Pinto et al. [6] (2023)	Survey of ML-IDS for critical infrastructure	Secondary	Till 2022	✗	✗	✗	✗
Our Taxonomic Review (2026)	ICS/IIoT dataset taxonomic review with decision aid	Primary	Till Q1 2026	✓	✓	✓	✓

✓ indicates the capability is provided; ✗ indicates it is not. Datasets Focus denotes whether datasets are the primary or a secondary object of the work. Coverage gives the latest dataset-release year each work reaches. Doc. Score = an explicit per-dataset documentation-completeness score. Use-Case Rubric = a structured dataset-selection decision aid. ATT&CK-ICS Map = mapping of dataset attacks to MITRE ATT&CK for ICS tactics. Use-Case Ranking = per-use-case dataset ranking. Mitseva [10] cites, and Koay [5] mentions, the MITRE ATT&CK for ICS matrix, but neither maps datasets to its tactics.

**Table 2:** Datasets used in ICS-IDS research.

Sr	Name	OT	Publisher	Year	Ref.
01	SWaT	Yes	iTrust Centre at Singapore University of Technology and Design	2015	[19,20,21]
02	WADI	Yes	iTrust Centre at Singapore University of Technology and Design	2017	[22,23]
03	EPIC	Yes	iTrust Centre at Singapore University of Technology and Design	2018	[24]
04	BATADAL	Yes	iTrust Centre, Technion, KIOS	2018	[25]
05	S317	Yes	iTrust Centre at Singapore University of Technology and Design	2017	[26]
06	MSU-GP	Yes	Power/Energy Lab, Mississippi State University	2014	[27,28]
07	MSU-PWR	Yes	Power/Energy Lab, Mississippi State University	2014	[29,30,31]
08	ICS-Flow	Yes	RISE Institute, Mälardalen University, Sweden	2023	[11]
09	Lemay	Yes	École Polytechnique de Montréal	2016	[32]
10	Electra	Yes	Department of Computer Engineering, University of Murcia, Spain	2019	[33]
11	Rodofile	Yes	Queensland University of Technology, Australia	2017	[34,35]
12	WUSTL-IIoT	Yes	CSE, McKelvey Engineering School, Washington University	2021	[36,37,38]
13	Edge-IIoTset	Yes	Department of CS, University of Guelma, Algeria	2022	[39]
14	HIL-WDT	Yes	University Campus Bio-Medico of Rome, Italy	2021	[40]
15	X-IIoTID	Yes	University of New South Wales, Australia	2022	[41]
16	TEP	Yes	ONR, Pacific Science and Engineering Group	2017	[42]
17	TLIGHT	Yes	Department of Computer Science, University of Hong Kong	2017	[43]
18	IUNO	Yes	German Research Center for AI (DFKI), Kaiserslautern	2019	[44,45]
19	ICS-NAD	Yes	Zhejiang University	2026	[46]
20	ICS-ADD	Yes	University of Genoa, Project RAISE	2024	[47]
21	HiTar	Yes	Tunisia Polytechnic, University of Carthage	2025	[48]
22	EDS	Yes	Zhejiang University	2024	[49]
23	HAI	Yes	Affiliated Institute of ETRI, South Korea	2020	[50,51]
24	KDD-CUP99	No	University of California, Information and CS Department	1999	[52]
25	NSL-KDD	No	University of New Brunswick, Canada	2009	[53]
26	UNSW-NB15	No	University of New South Wales, Australia	2015	[54]
27	CICIDS	No	University of New Brunswick, Canada	2017	[55]
28	ISCX	No	University of New Brunswick, Canada	2012	[56]
29	BoT-IoT	No	Australian Centre for Cyber Security	2019	[57]

**Table 3:** Selected datasets for comparative study.

Sr	Name	Domain	Environment	Citations (Google Scholar)	Citations (Scopus)	Availability
01	SWaT [19,20,21]	Water Treatment	Physical Testbed	1347	960	Request
02	WADI [22,23]	Water Distribution	Physical Testbed	790	564	Request
03	EPIC [24]	Electrical Power Grid	Physical Testbed	119	68	Request
04	BATADAL [25]	Water Distribution	Simulation	337	236	Public
05	S317 [26]	Water Treatment	Physical Testbed	56	35	Request
06	MSU-GP [27,28]	Gas Pipeline	Physical Testbed	367	246	Public
07	MSU-PWR [29,30,31]	Electrical Power Grid	Physical Testbed	579	408	Public
08	ICS-Flow [11]	Manufacturing	Simulation	115	59	Public
09	Lemay [32]	Electrical Power	Simulation	179	n/a	Public
10	Electra [33]	Railway	Real Application	159	108	Restricted (1)
11	Rodofile [34,35]	Mining Refinery	Physical Testbed	41	26	Public
12	WUSTL-IIoT [36,37,38]	Water Storage/IIoT	Physical Testbed	247	163	Public
13	Edge-IIoTset [39]	IoT/IIoT	Physical Testbed	1306	895	Public
14	HIL-WDT [40]	Water Distribution	Physical Testbed (4)	117	82	Public
15	X-IIoTID [41]	IIoT	Physical Testbed	344	234	Public
16	TEP [42]	Chemical Process	Simulation	169	n/a	Public
17	TLIGHT [43]	Traffic Light Control	Simulation	23	16	Restricted (2)
18	IUNO [44]	Water Storage	Physical Testbed	191	118	Restricted (3)
19	ICS-NAD [46]	Power Generation and STP	Real Application	3	1	Public
20	ICS-ADD [47]	Water Treatment	Simulation	59	34	Public
21	HiTar [48]	Manufacturing/IIoT	Simulation	5	4	Request
22	EDS [49]	Ethanol Distillation	Physical Testbed	41	34	Public
23	HAI [50,51]	Power Generation	Physical Testbed + HIL	80	46	Public

(1) The Electra dataset [33] was released, but no current public repository is found as of 2026. (2) They published in [43] a system specification only for TLIGHT, and no downloadable dataset is found. (3) The IUNO dataset [44] was released as part of the BMBF IUNO project, and no public repository was found as of 2026. (4) HIL-WDT can be considered a hybrid of a physical testbed plus a simulated MiniCPS. Citation counts were retrieved in June 2026 for each dataset’s primary reference from both Google Scholar and Scopus. Lemay and TEP have no Scopus entry (a workshop paper without a DOI, and a Harvard Dataverse dataset record, respectively).

**Table 4:** Testbed configurations of the surveyed datasets.

Dataset	Controllers	Field Devices (Sensors, Actuators, Equipment)
SWaT	6 Rockwell PLCs; 6 Schneider remote-I/O; HMI, SCADA, Historian;	Sensors and actuators across the 6 stages
WADI	3 Rockwell PLCs; 2 Schneider RTUs (Modbus TCP); Moxa gateways	Tanks, pumps, motorized and solenoid valves, flow meters, pressure transducers, pH/conductivity/turbidity analyzers
EPIC	PLCs, IEDs (with relays), switches, SCADA, historian	Motor-driven generators, PV panels, battery, and load
BATADAL	9 PLCs (PLC1 to PLC9) + SCADA; RTUs, smart meters	1 reservoir, 7 tanks, 11 pumps (5 stations), 4 valves
S317	SWaT testbed (capture from SWaT)	SWaT sensors/actuators (PCAP + Historian)
MSU-GP	SCADA master (MTU) + RTU	Pressure, flow, pump/solenoid states
MSU-PWR	Distance-protection relays (IEDs); PMUs/synchrophasors	Generators, transmission lines, breakers, relay + Snort logs
ICS-Flow	PLC	Sensors, actuators, valves, tanks, conveyor
Lemay	ScadaBR MTUs; Modbus_tk RTU emulators	Emulated source, breakers, voltage measurements
Electra	5 PLCs (1 master, 4 slaves); SCADA, switch, firewall;	Substation equipment; sensor and actuator logs
Rodofile	Siemens S7-300 master + 3x S7-1200 slaves; HMI; switches, hubs	Conveyor, wash tank, pipeline reactor
WUSTL-IIoT	Schneider Electric PLC	Water tank, level sensors, turbidity sensor, pumps, valve, alarm, HMI, historian
Edge-IIoTset	No OT PLCs; ONOS SDN controller, ThingsBoard, MQTT brokers	More than 10 IoT/IIoT devices, heterogeneous sensors
HIL-WDT	PLC(s) in hardware-in-the-loop	Real: 5 tanks, 20 solenoid valves, 4 pumps, 5 pressure sensors; simulated (MiniCPS): 3 tanks, 2 pumps, 4 flow sensors, 2 valves
X-IIoTID	PLC-related components; edge gateway; Web-SCADA/API	Field-tier sensors, actuators, local clients
TEP	None (process simulation)	Simulated process variables (no physical hardware)
TLIGHT	Siemens S7 PLC (polled via libnodave)	Pedestrian-request switches (inputs); traffic lights (outputs)
IUNO	Siemens S7 PLCs	2 water containers, centrifugal pump, solenoid valve, level/flow/pressure/temperature sensors
ICS-NAD	3 ICS vendors: ABB (thermal), Siemens (sewage), Schneider (sewage); subset of a larger 10-vendor site	Thermal-power (reheaters, burners) and sewage (sedimentation, filtration) equipment
ICS-ADD	ScadaBR (SCADA/HMI) + OpenPLC (PLC) on VMs	2 tanks, pumps; pfSense firewall, switch (SPAN), OSSIM SIEM, Suricata NIDS
HiTar	Controllers + workstations via AREZZO simulator	RFID reader/writer devices, shuttle stop-and-go elements
EDS	Siemens PLC; HMI, supervisory	Sensors, actuators; distillation with 3 control loops (temperature, flow, level)
HAI	Emerson Ovation DCS (boiler); GE Mark VIe DCS (turbine); Siemens S7-300 PLC (water-treatment); data acquisition via Siemens S7-1500 + ET200	GE turbine, Emerson boiler, FESTO MPS water-treatment, coupled via dSPACE SCALEXIO HIL

**Table 5:** Basic aspects across the studied datasets.

Sr	Dataset	Environment	Features	Format	OT Protocols in Testbed
01	SWaT	Physical Testbed	P/N	CSV/PCAP	EtherNet/IP
02	WADI	Physical Testbed	P	CSV	EtherNet/IP, Modbus TCP
03	EPIC	Physical Testbed	P/N	CSV/PCAP	IEC 61850 (GOOSE, MMS), Modbus TCP
04	BATADAL	Simulation	P	CSV	No protocols
05	S317	Physical Testbed	P/N	CSV/PCAP	EtherNet/IP
06	MSU-GP	Physical Testbed	P/N	CSV	Modbus RTU
07	MSU-PWR	Physical Testbed	P/N	CSV	IEEE C37.118
08	ICS-Flow	Simulation	P/N	CSV/PCAP	Modbus TCP
09	Lemay	Simulation	N	CSV/PCAP	Modbus TCP
10	Electra	Real Application	N	CSV, PCAP	Modbus, S7Comm, OPC
11	Rodofile	Physical Testbed	P/N	CSV/PCAP	S7Comm, DNP3
12	WUSTL-IIoT	Physical Testbed	N	CSV	Modbus TCP
13	Edge-IIoTset	Physical Testbed	P/N	CSV/PCAP	Modbus TCP, MQTT
14	HIL-WDT	Physical Testbed	P/N	CSV/PCAP	Modbus TCP
15	X-IIoTID	Physical Testbed	N	CSV	Modbus TCP, MQTT, CoAP
16	TEP	Simulation	P	R-Data	N/A
17	TLIGHT	Simulation	P	Unspecified	S7Comm
18	IUNO	Physical Testbed	P/N	Unspecified	OPC UA
19	ICS-NAD	Real Application	N	CSV/PCAP	Modbus, S7Comm, ABB (TCP)
20	ICS-ADD	Simulation	N	CSV/PCAP	Modbus TCP
21	HiTar	Simulation	N	Unspecified	Modbus TCP
22	EDS	Physical Testbed	P/N	CSV	S7Comm
23	HAI	Physical Testbed + HIL	P	CSV	OPC UA

**Table 6:** Mapping of dataset attack types to MITRE ATT&CK for ICS tactics.

Dataset	Attacks Mentioned/Normalized Attack Classes	MITRE ATT&CK for ICS Tactic Mapping
SWaT	False data injection; sensor spoofing; actuator manipulation; false process-state reporting; overflow/underflow; pump stress; wrong dosing	Impair Process Control; Inhibit Response Function; Impact
WADI	False data injection; sensor/actuator manipulation in water distribution	Impair Process Control; Inhibit Response Function; Impact
EPIC	False data injection; malicious/nuisance tripping; power interruption; physical damage; economic impact; malware-related disturbance	Execution; Impair Process Control; Inhibit Response Function; Impact
BATADAL	Pump/valve manipulation; tank-level manipulation; sensor falsification; replay/offset of process values	Impair Process Control; Inhibit Response Function; Impact
S317	Reconnaissance; DoS/SYN flooding; Layer-1 DoS; false data injection/process manipulation	Discovery; Inhibit Response Function; Impair Process Control; Impact
MSU-GP	Reconnaissance; command injection; response/measurement injection; DoS	Discovery; Execution; Impair Process Control; Inhibit Response Function; Impact
MSU-PWR	Data injection; remote tripping; relay setting change; control/relay manipulation	Execution; Impair Process Control; Inhibit Response Function; Impact
ICS-Flow	Port scan; IP scan; DDoS; MitM-based false data injection; replay	Discovery; Collection; Impair Process Control; Inhibit Response Function; Impact
Lemay	Polite/loud reconnaissance; data exfiltration; replay; Modbus command injection/manipulation	Discovery; Collection; Execution; Impair Process Control; Inhibit Response Function
Electra	Function-code recognition; Modbus/S7 read and write attacks; response modification; command modification; error response manipulation; replay	Discovery; Collection; Execution; Impair Process Control; Inhibit Response Function
Rodofile	PLC memory/register manipulation; turning sub-processes on/off; conveyor-direction change; wash-tank mode manipulation; reactor-threshold manipulation; emergency stop; global reset	Execution; Impair Process Control; Inhibit Response Function; Impact
WUSTL-IIoT	Reconnaissance; command injection; DoS; backdoor	Discovery; Execution; Persistence; Command and Control; Impair Process Control; Inhibit Response Function
Edge-IIoTset	DoS/DDoS; information gathering; port scanning; OS fingerprinting; vulnerability scan; MitM (DNS/ARP spoofing); XSS; SQL injection; file upload; backdoor; password cracking; ransomware	Discovery; Collection; Initial Access; Execution; Persistence; Command and Control; Inhibit Response Function; Impact
X-IIoTID	Reconnaissance; weaponization; exploitation; lateral movement; command and control; exfiltration; tampering; crypto-ransomware; ransom DoS (RDoS)	Discovery; Initial Access; Execution; Persistence; Lateral Movement; Collection; Command and Control; Impair Process Control; Inhibit Response Function; Impact
HIL-WDT	MitM (ARP poisoning); DoS (TCP flood, ICMP flood, LAND); scanning (SYN, FIN, NULL, XMAS); physical attacks (water leaks from manual valves; sensor failures; pump failures)	Discovery; Collection; Impair Process Control; Inhibit Response Function; Impact
TEP	Process faults/anomalies rather than explicit cyber-attacks	Not directly MITRE-mappable; can only be loosely related to process anomaly/fault-detection evaluation, not cyber-attack tactics
TLIGHT	False data injection using Snap7; PLC memory/address modification; traffic-light state manipulation	Execution; Impair Process Control; Impact
IUNO	Reconnaissance/scanning; malicious OPC UA response/measurement manipulation; covert PLC/process deviation with apparently normal reported values	Discovery; Impair Process Control; Inhibit Response Function; Impact
ICS-NAD	Reconnaissance; DoS/DDoS; false data injection; MitM	Discovery; Collection; Impair Process Control; Inhibit Response Function; Impact
ICS-ADD	DNS tunneling C2; port scanning; password brute-forcing; Modbus scanning; ARP spoofing/MitM; Modbus FDI; DoS	Discovery; Initial Access; Execution; Persistence; Command and Control; Collection; Impair Process Control; Inhibit Response Function; Impact
HiTar	Probing; R2L; U2R; DoS	Discovery; Initial Access; Privilege Escalation; Execution; Inhibit Response Function; Impact
EDS	Information leakage; replay attack; command injection; sensor data tampering; control parameter tampering; multi-point attack; physical attack	Collection; Execution; Impair Process Control; Inhibit Response Function; Impact
HAI	False data injection (set-point and sensor manipulation); control-parameter tampering; actuator override; coordinated multi-loop attacks; stealthy attacks (HAI 22.04+)	Impair Process Control; Inhibit Response Function; Impact

**Table 7:** Feature counts, instance counts, and class imbalance of the selected datasets.

Sr	Dataset	Features	Instances	Anomaly %	Imbalance Ratio ^(a)^
01	SWaT	51	946,722	12%	7.3:1
02	WADI	123	1,221,372	5.8%	16.3:1
03	EPIC	291/390	5222	Not reported	Not reported
04	BATADAL	43	15,027	14.35%	7.5:1
05	S317	28 ^(b)^	117,003 ^(b)^	0.14% ^(b)^	691:1 ^(b)^
06	MSU-GP	26	274,627	22%	3.5:1
07	MSU-PWR	128	78,377 (binary var.)	68%	0.5:1
08	ICS-Flow	50	45,719	19.7%	4.07:1
09	Lemay	Not reported	912,054 ^(b)^	0.89% ^(b)^	111:1 ^(b)^
10	Electra	10	41,641 (Modbus)/2,813,234 (S7Comm)	5.2% (Modbus)/1.42% (S7Comm)	18.2:1 (Modbus)/69.4:1 (S7Comm)
11	Rodofile	8	1.67 M	36.91% ^(b)^	1.71:1 ^(b)^
12	WUSTL-IIoT	41	1,194,464	7.3%	12.7:1
13	Edge-IIoTset	61	2,219,201	44%	1.3:1
14	HIL-WDT	55	7747	15.85%	5.31:1
15	X-IIoTID	59	820,834	49%	1.06:1
16	TEP	52	15,330,000	83.5% ^(b)^	0.20:1 ^(b)^
17	TLIGHT	10	2800/8000	Not reported	Not reported
18	IUNO	12	4910	14.3%	6.0:1
19	ICS-NAD	60	20,010,682 ^(b)^	49.99% ^(b)^	1.0:1 ^(b)^
20	ICS-ADD	Not reported	Not reported	Not reported	Not reported
21	HiTar	39	15,842	28.5%	2.5:1
22	EDS	47	843,321	41.9% ^(b)^	1.39:1 ^(b)^
23	HAI	78	1,323,608 ^(b)^	0.68% ^(b)^	147:1 ^(b)^

^(a)^ Normal-to-anomaly ratio = (100 − A%)/A%: 1. ^(b)^ Extracted by the authors from the released dataset files because the value is not stated in the original paper; the per-dataset notes that follow give the provenance of each marked entry. Lemay instance, anomaly, and imbalance values were extracted from the eleven released labeled captures; their entry counts match Table 1 of the source, and the unlabeled covert-channel exfiltration captures are excluded. Electra ships two subsets (Modbus and S7Comm). The instance counts are the deduplicated per-subset totals from Table 8 of [33]; the anomaly percentage and imbalance ratio are the as-generated class proportions from Tables 5 and 7 of [33], which reflect the dataset’s natural class balance (deduplication removes the highly repetitive normal traffic). Rodofile’s anomaly percentage and imbalance ratio were extracted from the released labeled master capture; the attack traffic is predominantly flooding, so the process-manipulation attacks are a very small fraction of the labeled packets. EDS’s anomaly percentage and imbalance ratio were computed from the released labeled files (652,855 records: 379,465 normal and 273,390 attack); the primary paper reports 843,321 records sampled over 117.1 h, including 72,965 from 10 h of normal operation. IUNO comprises three OPC UA captures. The Features, Instances, Anomaly %, and Imbalance Ratio in Table 7 are the values reported by the dataset’s authors for the OPC UA capture (Data Set 1) in [44] (4910 packets, 702 malicious → 14.3% anomalous; 6.0:1 normal-to-attack); 12 process features were engineered there and 10 were selected for classification. ICS-NAD’s instance, anomaly, and imbalance values were computed from the released Siemens subset of the labeled_CSV directory (20,010,682 labeled flow records: 10,007,776 benign and 10,002,906 attack; 19,050 rows without a label column were excluded); the ABB and Schneider subsets were not included. Attack traffic is predominantly flooding. ICS-ADD is released as a forensic evidence collection (a single SPAN packet capture plus SIEM and SCADA event logs) rather than a labeled feature dataset, so it carries no per-record benign/attack labels or feature schema; its Features, Instances, Anomaly %, and Imbalance Ratio therefore remain Not reported. The released files comprise 215,456 captured packets over an approximately six-minute window, 2339 SIEM events (including 102 Suricata NIDS alarms), and 78 ScadaBr point-value changes spanning a single Modbus false-data-injection window. For HAI, Table 7 statistics are reported for the HAI 21.03 release (78 monitored tags; 1,323,608 one-second records across the released train and test files, of which 8947 are attack-labeled); the anomaly percentage and imbalance ratio were computed by the authors from these files. Later HAI releases (22.04, 23.05, and the HAIEnd companion) raise the tag count to 86/225 but retain the same sparsely attacked design. SWaT and WADI counts correspond to the iTrust release files accessed 14 May 2026 [21]; published totals vary across release iterations. The S317 row uses the CISS 2019 (CISS2019.A1) release on the SWaT testbed: 117,003 one-second records with 169 attack-labeled rows (0.14% anomalous, 691:1), extracted from the released combined file (the raw per-day files total 136,805 records). BATADAL [25] releases three partitions; Training Dataset 1 (8761 samples) is attack-free by design. The 14.35% anomaly rate in Table 7 is computed over the attack-labeled partitions (Training 2 + Test, 6266 samples, ≈899 attack hours); over the full released corpus, including the attack-free baseline, it is 5.98%.

**Table 8:** Attack generation, realism, imbalance, and diversity of the selected datasets.

Sr	Dataset	Generation/Evidence	Realism	Imbalance ^(a)^	Diversity ^(b)^
01	SWaT	Manual documented attack scenarios; tools Wireshark, Ettercap, Aircrack-ng, Scapy	Injected	Moderate	Moderate
02	WADI	Manual process manipulation; tools NR	Injected	Severe	Moderate
03	EPIC	Smart-grid attack scenarios; tools NR	Injected	NR ^(c)^	Moderate
04	BATADAL	epanetCPA/EPANET attack simulation	Synthetic	Moderate	Moderate
05	S317	S3 attacker-team tools/scripts; not fully enumerated	Injected	Severe	Moderate
06	MSU-GP	Custom Modbus attack scenarios; tools NR	Injected	Mild	Diverse
07	MSU-PWR	Attack/event scenarios; Snort is logging/IDS	Injected	Mild	Moderate
08	ICS-Flow	Scripted scan, DoS/DDoS, replay, MitM-FDI; ICSFlowGenerator for extraction	Synthetic	Moderate	Diverse
09	Lemay	SCADA sandbox; Modbus traffic and exploit/command scenarios	Synthetic	Severe	Diverse
10	Electra	Wireshark capture; Python/Scapy parsing; attacker node for injected attacks	Real + Injected	Severe	Diverse
11	Rodofile	Python/Snap7-based PLC memory/register manipulation on physical S7Comm testbed	Injected	Mild	Moderate
12	WUSTL-IIoT	Backdoor, command injection, DoS, reconnaissance; tools not fully specified	Injected	Severe	Diverse
13	Edge-IIoTset	Reported IoT/IIoT attack tools/scripts for scan, brute force, web, malware, DoS	Injected	Mild	Diverse
14	HIL-WDT	Cyber and physical scenarios: MitM, flood, scan, leak, failure; tools partly NR	Injected	Moderate	Diverse
15	X-IIoTID	Lifecycle IIoT scenarios; Zeek/OSSEC are parsing/alert tools	Injected	Mild	Diverse
16	TEP	MATLAB/Simulink TEP faults; not explicit cyberattacks	Synthetic	Mild ^(d)^	N/A ^(d)^
17	TLIGHT	Snap7 alteration of selected Siemens PLC addresses	Synthetic	NR ^(c)^	Moderate
18	IUNO	OPC UA packet/application attacks and covert physical deviation	Injected	Moderate	Moderate
19	ICS-NAD	20 deliberate ICS attacks: recon, DoS/DDoS, FDI, MitM; Nmap, Hping3, Netwox, Arpspoof	Real + Injected	Mild	Diverse
20	ICS-ADD	Cyber Kill Chain-style pentest sequence; OSSIM/Suricata are monitoring tools	Synthetic	NR ^(c)^	Diverse
21	HiTar	AREZZO simulator logs; attack_labelling.sh; no live attack tool	Synthetic	Mild	Diverse
22	EDS	snap7/Python acquisition; leakage, replay, command injection, tampering, physical	Injected	Mild	Diverse
23	HAI	Automated attack tool; 38 to 58 attacks across HAI releases	Injected	Severe	Moderate

^(a)^ Imbalance degree follows Table 7: (Mild ≤ 4:1; Moderate = 4:1 to 9:1; Severe ≥ 10:1). ^(b)^ Attack diversity is defined by the number of distinct MITRE ATT&CK for ICS tactics a dataset exercises (Table 6): (Mild ≤ 2 tactics; Moderate = 3–4 tactics; Diverse ≥ 5 tactics). For TEP, the value is marked N/A because the dataset is primarily a process-fault dataset. ^(c)^ NR: not reported or not derivable from author or dataset documentation. ^(d)^ TEP reports process faults, not labeled cyber-attacks; Table 7 anomaly percentage and imbalance ratio are derived by treating the 20 fault types as one anomalous class and applying the documented fault-onset times, which yields 83.5% anomalous (0.20:1, rated Mild).

**Table 9:** Use-case rubric scores per dataset (sorted by percentage of achievable maximum).

Rank	Dataset	Realism	Divers.	Imb.	Doc.	Repro.	Total/Max	%
1	Edge-IIoTset	1	3	3	3	3	13/15	86.7
2	ICS-NAD	2	3	3	2	3	13/15	86.7
3	X-IIoTID	1	3	3	3	2	12/15	80.0
4	MSU-PWR	1	2	3	3	2	11/15	73.3
5	ICS-Flow	0	3	2	3	3	11/15	73.3
6	Rodofile	1	2	3	2	3	11/15	73.3
7	HIL-WDT	1	3	2	2	3	11/15	73.3
8	MSU-GP	1	3	3	2	2	11/15	73.3
9	EDS	1	3	3	2	2	11/15	73.3
10	HAI	1	2	1	3	3	10/15	66.7
11	SWaT	1	2	2	3	2	10/15	66.7
12	WUSTL-IIoT	1	3	1	3	2	10/15	66.7
13	ICS-ADD	0	3	—	2	3	8/12	66.7
14	BATADAL	0	2	2	2	3	9/15	60.0
15	HiTar	0	3	3	1	2	9/15	60.0
16	Lemay	0	3	1	2	3	9/15	60.0
17	WADI	1	2	1	3	1	8/15	53.3
18	Electra	2	3	1	2	0	8/15	53.3
19	TEP	0	0	3	2	3	8/15	53.3
20	EPIC	1	2	—	1	2	6/12	50.0
21	IUNO	1	2	2	2	0	7/15	46.7
22	S317	1	2	1	1	2	7/15	46.7
23	TLIGHT	0	2	—	2	0	4/12	33.3

TEP is scored 0 for attack diversity because it is primarily a process-fault/anomaly dataset, not an explicit cyber-attack dataset. The score should be read as a decision-support score, not as an absolute scientific ranking. Domain and protocol fit may override the total score in specialized studies. A renormalization is performed to exclude the Imbalance where unreported, so the total is 12 for them.

**Table 10:** Dataset types, their defining criteria, and member datasets.

Category	Defining Criterion	Member Datasets	*n*
Process-aware ICS/SCADA	Real or simulated industrial process with control-network or physical-process data (water, power, and other sectors)	SWaT, WADI, BATADAL, S317, HIL-WDT, IUNO, ICS-ADD, EPIC, MSU-PWR, Lemay, ICS-NAD, HAI, MSU-GP, Rodofile, ICS-Flow, EDS, Electra, TLIGHT	18
Hybrid IIoT–ICS	Industrial-IoT testbeds layered over a physical process	WUSTL-IIoT, X-IIoTID	2
Generic IIoT/IoT	Broad IoT/IIoT intrusion datasets that are not process-aware	Edge-IIoTset, HiTar	2
Process-fault/anomaly	Process fault-detection data without deliberate cyber-attacks	TEP	1

## Data Availability

The data discussed in this study are derived from publicly available and request-access datasets. No new primary data were generated by the authors. The 23 datasets analyzed in this review are listed in Table 3, along with their availability. For each dataset, the access link (repository, archive, or request page) and the access date are provided in Supplementary Table S1; the datasets were accessed in May 2026 and their availability and links were re-verified in June 2026.

## Supplementary Materials

The following supporting information can be downloaded at: https://www.mdpi.com/article/10.3390/s26134099/s1, The Supplementary Material S1 (Dataset Overview) provides the detailed per-dataset descriptions for the 23 datasets. It also comprises the supporting information that can be downloaded at the journal website, Table S1: Dataset availability and access (per-dataset availability status, access link or request page, and access date for the 23 datasets). References [21,64,65,66,67,68] are cited in the Supplementary Materials.
