# Supplementary material for: Intrusion Detection Datasets for IIoT and ICS: A Taxonomic Review with a Decision-Aid Scoring Rubric
Source: Sensors (Basel). 2026 Jun 27;26(13):4099. doi: 10.3390/s26134099 (PMC13363749; doi:10.3390/s26134099)
Supplement: Supplementary file 1 [file sensors-26-04099-s001.zip › sensors-4359554-supplementary.pdf]

sensors MDPI

SUPPLEMENTARY MATERIAL S1

Dataset Overview (Descriptions and Repositories)

This file (Supplementary Material S1) provides, for the 23 datasets analyzed in the main manuscript (Intrusion Detection Datasets for IIoT and ICS, Manuscript ID sensors), both the detailed per-dataset descriptions (corresponding to Section 4.3 of the main text) and a consolidated dataset availability and repository table (Table S1). The dataset subsections are numbered S1.1 to S1.23 and follow the main-text ordering (Section 4.3); cross-references to tables, sections, and other figures refer to the main manuscript. Citation numbers in this file (for example [34]) refer to the reference list of the main manuscript.

S1.1. SWaT Dataset

The iTrust Center for Research in Cyber Security at Singapore University of Technology and Design (SUTD) has developed datasets that contain both normal and malicious samples [21]. The SWaT testbed is a scaled-down version of a modern water treatment facility. It consists of multiple components forming a six-stage water treatment process. The system can treat and purify 19 liters of water per minute [19]. The system architecture and interfaces of the testbed are available on the iTrust testbeds webpage (https://www.sutd.edu.sg/itrust/itrust-labs/testbeds). The SWaT dataset was collected over continuous operation for eleven days. It recorded 7 days of normal operations and 4 days during which various attacks were executed. The CSV process dataset contains readings from 51 sensor/actuator tags. Network traffic is stored in separate packet-level files [20].

iTrust researchers performed 41 attack scenarios and manually recorded the time of the attacks, the sensor or actuator targeted, and any physical consequences. The network traffic was collected simultaneously on the network (EtherNet/IP) and on the corporate LAN, resulting in packet-level PCAP files and sensor value CSV logs [21].

S1.2. WADI Dataset

The Water Distribution testbed is the downstream extension of SWaT [22]. The system architecture and interfaces of the testbed are available on the iTrust testbeds webpage (https://www.sutd.edu.sg/itrust/itrust-labs/testbeds), and consists of 3 Rockwell PLCs using EtherNet/IP and 2 Schneider RTUs using Modbus TCP, bridged through Moxa Modbus/Eth IP gateways. The first PLC controls the primary reservoir, the second the consumer tank, and the last the return water. The historian stores all process values with a 1-second sampling rate.

The dataset was acquired in normal operation and attack operation in 2017. 15 attacks were applied related to the malicious manipulation of valves, pumps, flow transmitters, level readings, quality readings, chemical dosing, consumer-supply valves, leakage paths, and booster pressure set points. Some attacks are coordinated or stealthy, e.g., draining elevated reservoirs while tricking the sensors to hide the actual impact [23].

S1.3. EPIC Dataset

As a cyber-physical systems testbed, the Electrical Power and Intelligent Control testbed was developed at SUTD iTrust to facilitate smart-grid security research. It contains four major subsystems starting from generation, along transmission, passing the micro-grid, and finally the smart home. It aims to facilitate experiments on cyber-physical attacks, the cascading effects, and the testing of attack-detection and defense solutions. The control system consists of PLCs, IEDs (Intelligent Electronic Devices), switches, SCADA, and historian functions [24].

The system architecture and interfaces of the testbed are available on the iTrust testbeds webpage (https://www.sutd.edu.sg/itrust/itrust-labs/testbeds), which comprises supervisory control, local control, protection, and network communication. The entire system is monitored by the SCADA workstation and is used for supervisory control, with protection and control functions accomplished by the IEDs. The EPIC paper mentions the use of WAGO PLCs for breaker control and generator sync logic, and Siemens SIPROTEC relays as IEDs. Communication is based on IEC 61850 as the primary standard, which facilitates the transfer of events and system data.

GOOSE and MMS are utilized in the IEC 61850 communication landscape to transfer data between IEDs and the SCADA workstation. GOOSE is used as the IEC 61850 mechanism for fast event/protection-related messaging, while MMS can be considered the IEC 61850 communication service for monitoring and controlling data exchange between supervisory entities and field/control devices [64]. The paper also mentions that communication between SCADA and PLCs can be wired or wireless, and that in some PLC configurations Modbus TCP/IP may be used in addition to IEC 61850. EPIC is capable of supporting a variety of attack scenarios applicable to smart-grid security. The authors outline four possible classes: power supply interruption, nuisance tripping, physical damage, and economic-advantage attacks. Two of these were demonstrated: a power supply interruption attack and a physical damage attack. EPIC captures realistic cyber-physical behavior of a physical smart-grid testbed in normal operation and selected attack scenarios.

S1.4. BATADAL Dataset

The Battle of the Attack Detection Algorithms dataset is the result of a community-wide competition to compare cyber-physical attack detection algorithms for water distribution networks. The medium-sized, real-world, C-Town water distribution system was controlled by programmable logic controllers (PLCs) and a SCADA system. Participants were given simulated SCADA observations and asked to identify whether the system was operating normally or under attack, with performance evaluated using classification accuracy and time to detect the intrusion [25].

The PLCs monitor and control pumps, tanks, and valves, and the SCADA system coordinates operations and stores PLC readings. Hence BATADAL is regarded as a simulated dataset. The datasets were created with epanetCPA, a MATLAB toolbox for designing cyber-physical attacks and simulating the hydraulic consequences with EPANET. The hydraulic simulation was run with a higher internal time resolution, and the SCADA

observations made available to participants were sampled at regular 1-hour intervals. The set of SCADA observations released includes 43 variables: 7 water-level variables in the tanks, 12 pressure variables at selected locations in the network, and 24 flow-status variables for pumps and the actuated valve.

Three datasets were released. Training Dataset 1 contained 8,761 hourly samples (~365 days) of normal operation without any cyber-attack. Training Dataset 2 contained 4,177 hourly samples (~6 months) and included seven attacks spanning approximately 492 attack-labeled hours; attack labels were provided for training and validation, but not all attacks were fully disclosed during the competition. The Test Dataset contained 2,089 hourly samples (~3 months) with seven additional attacks spanning approximately 407 attack-labeled hours, where attack information was withheld from participants during evaluation. In total, the corpus comprises 14 attack scenarios across the three datasets.

The attack scenarios include actuator activation and changes to actuator settings, and deception attacks affecting sensor, PLC, and SCADA communications. Examples include changing pump-control thresholds, reducing pump speed, maliciously activating pumps, manipulating tank-level signals, and concealing the effects of attacks. The original BATADAL dataset contains time-series sensor/actuator observations and does not contain network-level packet captures or PCAP files. This sets it apart from the later BATADAL 2.0 work that was created to provide a more detailed representation of industrial communication/network processes.

S1.5. S317 Dataset

The S317 dataset is from the SWaT Security Showdown (S3), an Industrial Control System security event. It was a gamified ICS education and research event where independent attacker teams from academia and industry attacked SWaT/WADI, while academic teams attempted real-time detection. According to Antonioli et al., a Capture-the-Flag event is an ICS-focused exercise composed of attacker teams, a live attack-defense phase, and data collection for later analysis [26]. The dataset includes three types of evidence gathered during the event: PCAPs of the network packets captured, Historian data from the process level, and documentation of the attack scenarios conducted by participants. The PCAP files enable researchers to analyze communication-level data such as protocol behavior, packet timing, and network anomalies, while the Historian data enables analysis of physical-process data and actuator states from the SWaT plant. Because of these features, the dataset can be used to assess intrusion detection techniques that combine cyber-network indicators with process-state changes [26].

The S317 entry in this review also covers the later editions of the SUTD/CISS Security Showdown generated on the same SWaT testbed, in particular the CISS 2019 (CISS2019.A1) release collected from 27 to 30 August 2019. That release provides a combined, attack-labelled record set of 117,003 one-second samples across 28 process sensors together with the associated actuator and stage signals, and a list of 187 launched attacks; its feature, instance, anomaly, and imbalance values in Table 7 are extracted from these

released files. We consider these CISS editions under the single S317 entry because they share the same testbed, protocols, and security-showdown methodology.

S1.6. MSU-GP Dataset

The MSU Gas Pipeline dataset was released by Morris and Gao (2014) at Mississippi State University as one of two companion datasets derived from laboratory-scale physical ICS testbeds that use the Modbus application-layer protocol [27]. The gas pipeline testbed implements a supervisory control system for a small-scale gas-flow process, with a SCADA master station (MTU) communicating over serial Modbus with a remote terminal unit (RTU) that monitors and actuates process variables such as pressure, flow, and pump/solenoid states. While the testbed is not an operational natural-gas pipeline, it physically reproduces the cyber-physical interactions characteristic of gas pipeline SCADA communication and control [27].

The testbed adopts a typical master–slave architecture, with the master station periodically polling the RTU. The Modbus protocol provides no inherent authentication, integrity, or availability protections, leaving it vulnerable to attacks that alter commands, responses, measurements, or service availability. Morris and Gao's work documents datasets of process variables and network-traffic features captured during the execution of 28 attacks across the gas pipeline and water storage tank testbeds [27].

The dataset is distributed in ARFF format (a CSV-compatible format), with both a preprocessed feature dataset and a raw Modbus-frame dataset that enables independent feature engineering. Each record combines network-communication attributes, process-related measurements, and a ground-truth attack label, making the corpus suitable for supervised intrusion detection, both binary (attack vs. normal) and multiclass (attack-category) classification. Attack scenarios cover four high-level categories: reconnaissance, command injection, response or measurement injection, and denial-of-service. These threats either target the cyber layer by manipulating Modbus communication or affect the cyber-physical layer by altering the apparent or actual state of the gas pipeline process [27]. The widely used gas-pipeline subset evaluated in the IDS literature is the Turnipseed (2015) variant built on the same MSU testbed, which contains 274,627 labeled instances with refined randomness compared to the original release [28].

S1.7. MSU-PWR Dataset

This dataset was generated at the MSU Power and Energy Research Lab electric-power cyber-physical testbed by Adhikari, Pan, and Morris, with the raw logs subsequently formatted into ARFF/CSV releases through a collaboration with Borges and Beaver at Oak Ridge National Laboratory (ORNL) [29,31].

It simulates a simple transmission network, allowing researchers to observe the impact of cyber/operational events on both power-system measurements and operational/security logs [29].

The released dataset has been utilized in supervised and anomaly-based IDS studies because it offers labeled examples of normal, disturbance, and attack behavior in a power-system context. The dataset is composed of Synchrophasor/PMU measurements, relay

logs, network-event-monitor logs, control-panel logs, and Snort alert indicators, all of which are time-synchronized but heterogeneous. It consists of 15 sets of power-system scenarios, with 37 event scenarios in each set. The attack scenarios are remote tripping command, data injection, and changes in relay setpoints [29,65]. This structure is important for cyber-physical IDS research as it helps to differentiate between malicious cyberattacks and non-malicious power-system disturbances, which is essential for minimizing false alarms in real power-system monitoring.

The dataset is released in three sub-versions (binary, three-class, and multiclass). They are built from fifteen sets of 37 scenarios with 1% random sampling [29,30], and the figures reported in Table 7 correspond to the binary sub-dataset. This dataset has been used as a benchmark for hybrid IDS [29] and disturbance-versus-attack classification [30,66].

S1.8. ICS-Flow Dataset

The ICS-Flow dataset was introduced by Dehlaghi-Ghadim et al. (2023) and was created in a simulated ICS environment that represents a bottle-filling manufacturing process. This dataset includes process data and networking traffic. The released information includes raw packet captures, network flow data, and logs of process variables, which allow researchers to examine the behavior of both communications and the physical process under normal and abnormal conditions. The authors also developed the open-source tool ICSFlowGenerator, which allows the extraction of ICS-oriented flow features from raw network packets. In total, the final dataset includes over 25 million raw network packets, flow records, and logs of process-state variables [11].

Two categories of attacks are performed: reconnaissance and disruption (IP/port scanning, DoS/DDoS), and Modbus communication manipulation (replay of previously captured valid packets, alteration of Modbus register values) [11]. Because the dataset is released simultaneously at packet, flow, and process-variable granularity, it supports both supervised and unsupervised IDS evaluation across abstraction levels.

S1.9. Lemay Dataset

The Lemay/Fernandez dataset provides SCADA network traffic and was generated in a SCADA sandbox rather than collected from a real plant or testbed. To give the process values realism, the authors added an electrical-network simulation: each controller represents a small electrical network including an electrical source, breakers, and voltage measurements. We can consider this a software-emulated SCADA/Modbus dataset [32].

The captured data includes both benign and malicious Modbus packets in full capture, along with CSV label files for supervised machine-learning experiments. The normal traffic is primarily the deterministic polling of typical Modbus SCADA traffic, and some of the captures include simulated manual operations (Modbus write commands). The attack scenarios were executed live against the sandboxed SCADA network, rather than simply being injected into an already finished capture. The authors detail attacks that include targeting another RTU with a Metasploit MS08-netapi exploit, transferring files via a Meterpreter channel, Modbus fingerprinting using multiple read commands, and an

unauthorized Modbus WRITE_COIL command sent from an attacked RTU. The release also includes a separate Modbus covert-channel dataset [32].

The dataset does not fully represent a production SCADA network. The simulated RTUs share near-identical configurations, polling intervals are fixed within each capture, and the manual write operations are scripted rather than driven by a human operator. Only MTU-to-RTU traffic is captured; the link between RTUs and field devices lies outside the dataset.

S1.10. Electra Dataset

Electra is a relatively realistic ICS dataset compared to many laboratory-only or purely simulated datasets because it was generated from actual network traffic of an electric traction substation in the railway sector, as introduced by Perales Gómez et al. (2019). The system is not a typical railway signaling or rolling-stock system, but a railway traction-power system. The system architecture supports Modbus TCP, S7Comm, and OPC, while the released dataset is split into two protocol-specific subsets (Electra Modbus and Electra S7Comm) [33].

All normal and malicious network traffic was captured during data collection. Multiple PCAP files (about 20 GB) were generated by capturing mirrored network traffic using Wireshark, with the buffer size set to 200 MB. Once captured, the traffic was split into two sets: Modbus packets (TCP port 502), and S7Comm (TCP port 102). The authors then used Python and Scapy to parse the packet captures and extract packet-level features into CSV files. A variety of features was selected, including timestamp, src/dest MAC and IP address, request/response indicator, function code, error indicator, memory address, data value, and label. Multi-operation packets were split into one read or write operation per dataset instance [33].

Reconnaissance, false data injection, and replay attacks are represented in Electra. Specifically, the attacks include function-code recognition, read-attack traffic, write-attack traffic, modification of responses and commands, force-error attacks, and replay of previously observed valid packets. These attacks do not stem from truly uncontrolled adversary activity; they are actively executed in the substation environment by an attacker node. The dataset is not real incident traffic, but rather realistic industrial testbed traffic with executed/injected attacks.

A subsequent study by Pallakonda et al. (2025) tested a secure anomaly detection system that integrates machine-learning classifiers with hybrid AES/RSA encryption, protocol hardening, and threat-hunting techniques, employing the Electra Modbus subset. The model achieved good performance for binary and multi-class classification [67].

S1.11. Rodofile Dataset

The Rodofile dataset was produced at the Queensland University of Technology using a physical SCADA process-control testbed that simulates a simplified mining refinery plant. The plant comprises three processes (conveyor, wash tank, and pipeline reactor). The testbed architecture (Figure S1) includes a Siemens SIMATIC S7-300 Master and three S7-1200 slave PLCs, where each slave PLC is responsible for one process. The HMI is used

by the operator to monitor and control the simulated process and store historian logs that record the state of the control process during the experiment [34]. Additionally, there is an attacker workstation, a GPS clock, managed switches, and two extra hubs to capture traffic from the Master PLC and HMI viewpoints, as well as for dataset labeling.

The attacks do not exploit vulnerabilities in the S7Comm parser or its implementation. They leverage the lack of authentication and integrity in the protocol to write arbitrary values to PLC registers. This disrupts the timed sequencing and safe operation of the industrial process.

The released dataset includes network traffic and process logs. The attack dataset was recorded for about 9 hours and consists of 30 process runs, whereas the control dataset consists of about 8.5 hours of data during normal operation with 32 process runs. The attack data consists of 64 attack instances. There are packet-capture files and four process logs included for each dataset (Tank log, Conveyor log, Reactor log, and Master log). The dataset is thus useful for IDS research, as both S7Comm network traffic and process-state behavior can be analyzed. A major drawback, however, is that the process is small-scale, scripted, and modeled in a laboratory setting rather than a complete, production-scale mining or mineral-processing plant.

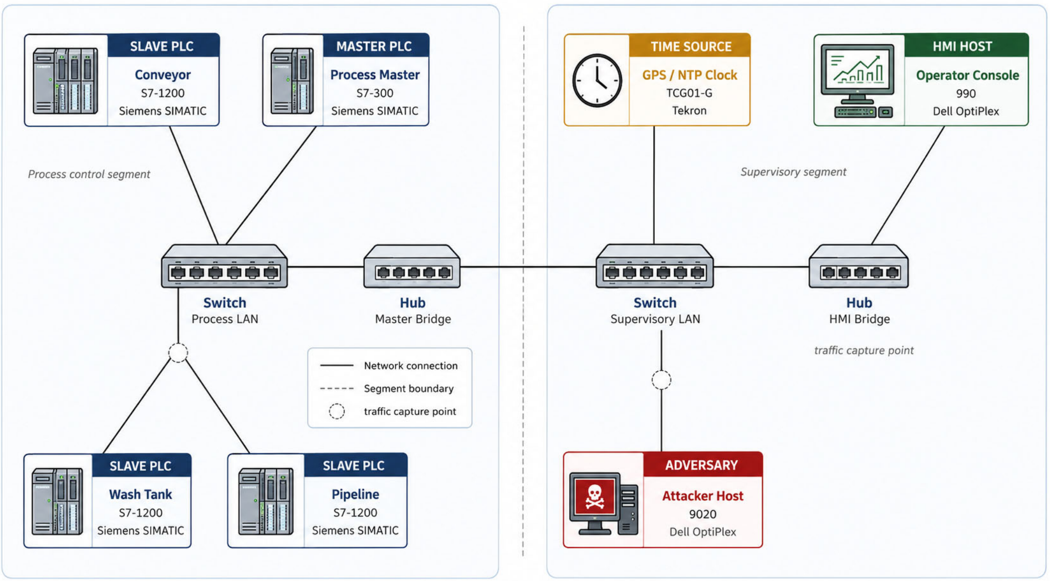

Figure S1. Testbed Interfacing Diagram Described by Rodofile et al. [34]

S1.12. WUSTL-IIoT Dataset

This dataset was produced by Zolanvari et al. from Washington University in St. Louis (WUSTL) for cybersecurity research in the Industrial Internet of Things. It was generated from an IIoT testbed designed to simulate an industrial plant and enable real cyberattacks against the system. The physical process consists of a water tank, water-level sensors, a turbidity sensor, pumps, a valve, alarm indicators, an HMI, historian/logging components, and a PLC. The testbed communicates with the control components via Modbus/TCP, and the PLC is based on a Schneider Electric controller [36]. The setup is therefore best described as a laboratory IIoT/ICS water-process testbed.

The released dataset consists of network flow records extracted from traffic collected during both normal operation and attack scenarios. The authors report collecting 2.7 GB of data in approximately 53 hours, followed by preprocessing. The released dataset has 1,194,464 instances and 41 selected network-flow features, of which 1,107,448 are normal and 87,016 are attack. The authors recommend removing certain features (such as start/end time or IP identifier/address) prior to modeling, as they can provide attack-specific information and reduce the ability to generalize to unseen data.

The dataset labels support both multi-class and binary intrusion-detection formulations. The original Traffic label identifies the traffic type, while binary classification can be obtained by mapping all attack traffic to one attack class and normal traffic to another. The main attack categories reported are command injection, denial-of-service, reconnaissance, and backdoor traffic. The overall attack proportion is less than 8%, with normal traffic representing about 92.72% of the dataset and total attack traffic about 7.28%. Among the attack samples, DoS dominates the distribution, while command injection and backdoor samples are comparatively rare. This deliberate imbalance reflects the authors' motivation to approximate a more realistic IIoT security setting, where attack events are much less frequent than normal traffic [36].

WUSTL-IIoT-2021 is therefore useful for evaluating IDS models in an IIoT/ICS context, especially under class-imbalance conditions. However, it remains a controlled laboratory dataset rather than production traffic. The released data are primarily preprocessed flow features rather than full raw packet/process datasets, and some features must be carefully removed to avoid leakage during model training.

S1.13. Edge-IIoTset Dataset

Ferrag et al. (2022) presented this dataset for IoT/IIoT intrusion detection research that can be evaluated using either centralized or federated machine learning. The dataset is captured through a specially designed IoT/IIoT testbed. It is structured into seven layers covering cloud computing, network function virtualization, blockchain, fog computing, software-defined networking, edge computing, and IoT/IIoT perception. This layered architecture was intended to depict a modern edge-cloud IoT/IIoT deployment and not a traditional SCADA-only architecture [39].

It has 2,219,201 labeled records: 1,246,500 normal and 972,701 attack. The authors first identified 1,176 raw features from various sources, including network traffic, logs, system resources, and alerts. They then selected 61 high-correlation features for machine learning. The generated files consist of normal and attack traffic in PCAP and CSV formats, along with selected CSV files ready for traditional ML and deep-learning experiments [39]. It may not be a classical OT/SCADA dataset, since it primarily focuses on heterogeneous IoT/IIoT edge and cloud traffic.

S1.14. HIL-WDT Dataset

The Hardware-in-the-Loop Water Distribution Testbed dataset was introduced by Faramondi et al. (2021) at the University Campus Bio-Medico of Rome, Italy. The testbed is a water distribution process within a hardware-in-the-loop architecture, in which a real

physical water distribution process is virtually interconnected with a simulated unit. This design allows the dataset to capture both the physical consequences of attacks and their corresponding network-level behavior.

The entire HIL-WDT process consists of eight tanks, five of which are actually implemented. The process is divided into four stages; the first stage is controlled by a real PLC, and the other stages are controlled by simulated PLCs. The PLC used in the testbed is a Modicon M340, and the communication protocol is Modbus TCP/IP.

Physical process data and network traffic data are included in the dataset. The historian records the physical measurements every second and saves them in CSV files. The network traffic is captured in PCAP files using Wireshark, and the extracted features are saved in CSV files. There are 41 features in the physical data, including tank levels, pump states, flow-sensor readings, and valve states. The network dataset includes 14 features such as src/dest IP and MAC addresses, ports, protocol, TCP flags, payload size, Modbus function code/value, and short-window packet counts.

The data are broken into four acquisitions of approximately 2 hours total. One acquisition is normal operation, and the others are attack scenarios. The authors specify 28 attack scenarios, including both cyber and physical attacks. Cyberattacks include man-in-the-middle attacks based on ARP poisoning, denial-of-service attacks (TCP flood, ICMP flood, LAND), and scanning attacks (SYN, FIN, NULL, XMAS). Physical attacks involve water leaks from manual valves, and sensor and pump failures. Importantly, some attacks impact only network traffic, some only the physical process, and some both. This renders HIL-WDT suitable for analyzing the correlation between cyber events and physical process anomalies.

S1.15. X-IIoTID Dataset

Al-Hawawreh et al. (2022) introduced the X-IIoTID dataset for IIoT. It is primarily designed to overcome the heterogeneity of IIoT systems, where different types of devices, communication patterns, and protocol families are present. The dataset was created using the Brown-IIoTbed testbed at UNSW Canberra, which combines legacy industrial devices and newer IoT, edge, cloud, and enterprise devices. The testbed consists of three tiers: edge, platform, and enterprise. The edge tier contains field devices, PLC-related components, sensors, actuators, an edge gateway, and local clients. The platform tier comprises a cloud application, an MQTT broker, and cloud storage. Finally, the enterprise tier encompasses the Web-SCADA/API, remote maintenance, and attacker machines [41].

The dataset is a collection of IIoT communication and system behavior from various sources. The authors gathered end-to-end network traffic, host-device logs, host-resource measurements, device-operational logs, and alert logs from OSSEC and Zeek. Zeek was used to generate connection logs for network feature extraction, and a combination of Python and batch scripts was used to parse, correlate, enrich, and label the data collected. The dataset comprises traffic and attack behavior with legacy and recent IIoT protocols, including Modbus, MQTT, CoAP, WebSocket, HTTP, SSH, DNS, ICMP, SMTP, TCP, UDP,

and ARP. Thus, X-IIoTID can be considered a heterogeneous IIoT intrusion dataset rather than a traditional single-protocol ICS dataset.

X-IIoTID offers a structured taxonomy of attacks comprising nine high-level attack classes: reconnaissance, weaponization, exploitation, lateral movement, command and control, exfiltration, tampering, crypto-ransomware, and ransom denial-of-service. These are further broken down into 18 attack sub-types, including generic scanning, vulnerability scanning, CoAP resource discovery, fuzzing, brute force, dictionary attack, malicious insider activity, reverse shell, man-in-the-middle, Modbus register reading, MQTT cloud-broker subscription, TCP relay, false-data injection, fake notification, crypto-ransomware, and RDoS. The final dataset totals 820,834 records, of which 421,417 are benign and 399,417 are malicious, and supports labeling at three levels of granularity: binary, nine-category, and eighteen-subtype classification.

One of the main advantages of X-IIoTID is that it facilitates more generalizable IIoT intrusion detection models. Its features are described as connectivity- and device-agnostic, meaning they are not restricted to a single vendor, device family, or application protocol. This makes the dataset better suited to evaluating IDS methods in heterogeneous IIoT environments than datasets restricted to a particular protocol, such as Modbus, S7Comm, or DNP3. The current version, however, does not address direct attacks such as false commands or false process data at the field-device level. It has very imbalanced minority attack classes, such as fake notifications and MitM. Therefore, X-IIoTID is strong for multi-protocol IIoT network/host intrusion detection, but it should not be presented as a complete replacement for physical-process ICS datasets.

S1.16. TEP Dataset

The Tennessee Eastman Process (TEP) dataset, released by Rieth et al. (2017), is a simulation-based industrial process dataset designed for anomaly detection evaluation. It is based on the Tennessee Eastman Process benchmark, a chemical process simulation widely used in fault detection and process monitoring research. The dataset is provided through Harvard Dataverse as "Additional Tennessee Eastman Process Simulation Data for Anomaly Detection Evaluation" [42] and is intended to support more reliable evaluation of anomaly-detection methods by providing multiple simulation replications with non-overlapping random-number-generator seeds, rather than the single training/testing run available in earlier TEP releases.

The dataset is organized into four R data files (fault_free_training, fault_free_testing, faulty_training, faulty_testing). Each file is loaded as an R dataframe with 55 columns, where 3 identifier columns (faultNumber, simulationRun, sample) and 52 TEP process variables constitute the features reported in Table 7. The faultNumber column identifies the process condition: fault-free data carry fault 0, and faulty data carry fault types numbered 1–20, for a total of 21 process conditions. The simulationRun column ranges from 1 to 500, identifying the replicate from which the row was generated. The sample column is the time index. Sampling follows the standard TEP rate of one observation every 3 minutes. Each training simulation has 500 samples over 25 hours of operation; each testing

simulation has 960 samples over 48 hours. In the faulty datasets, faults are introduced at one hour into the training simulations and at eight hours into the testing simulations, so the datasets support evaluation of both detection accuracy and detection delay after fault introduction [42].

This dataset is best characterized as a simulated chemical-process time series that includes process measurements under both normal and faulty operating conditions. It is highly suitable for process-level anomaly and fault-detection studies, but less suitable for evaluating network-based IDS models that require packet, flow, or protocol features.

S1.17. TLIGHT Dataset

Yau and Chow (2017) proposed the TLIGHT dataset to study the detection of anomalous events in PLCs using machine-learning techniques. Rather than a conventional network-traffic IDS dataset, TLIGHT is better understood as a PLC memory-state/event-log dataset generated from a simulated traffic-light control system implemented on a Siemens S7 PLC. The PLC I/O configuration maps pedestrian-request switches to PLC inputs and traffic lights to PLC outputs.

Data was collected by polling the relevant PLC memory addresses through a custom program built on libnodave (an open-source communications library for Siemens S7 PLCs). The values of the monitored inputs, outputs, timers, and memory bits were logged with timestamps, and non-binary values such as timer counts were converted into binary features for machine-learning processing. The paper identifies seven normal traffic-light operating states, each defined by a specific combination of timer and output values, which serve as the ground truth for the normal class.

Two experimental datasets were constructed using 10 features per record. Dataset 1 contains 2,800 records (560 training, 2,240 testing); Dataset 2 contains 8,000 records (1,600 training, 6,400 testing). Anomalous observations were generated by manipulating selected PLC address values with Snap7, an open-source suite of Ethernet communication tools for Siemens S7 PLCs. Yau and Chow report high classification accuracy with Decision Tree and Support Vector Machine classifiers from scikit-learn. The dataset has several documented limitations: the scenario is a small simulated traffic-light controller; the anomalies are scripted PLC-memory writes rather than realistic adversary behavior; the learning approach treats each observation independently and does not model temporal dependencies between states; and no publicly downloadable dataset repository is referenced, so subsequent works regenerate the data from the published system specification.

S1.18. IUNO Dataset

Duque Antón et al. (2019) proposed the IUNO/DFKI dataset to provide labeled training data for industrial intrusion detection. It was designed and built at the German Research Center for Artificial Intelligence (DFKI) in Kaiserslautern as part of the IUNO Insec project, funded by the German Federal Ministry of Education and Research (BMBF). The dataset is derived from a SCADA scenario with real industrial hardware in which Siemens S7 PLCs control a Festo Didactic MPS PA Compact Workstation (a laboratory training rig that emulates a water-tank filling and emptying process). Although the controllers are

Siemens S7 PLCs, the captured communication is OPC UA rather than S7Comm or Profibus, so the dataset is properly characterized as an OPC UA-based laboratory industrial-process dataset [44,45].

Three datasets were created, each consisting of normal process operation with introduced attacks. The first is the captured baseline traffic with synthetic malicious OPC UA messages added offline, and sensor/actuator values are manipulated to simulate false-data injection. The second introduces malicious behavior at the PLC/application layer: a malicious OPC UA client performs reconnaissance, and the PLC returns falsified process values in response. The third captures apparently normal PLC-reported data while the physical process is in fact abnormal, and uses side-channel measurements (sound, flow, and temperature) to expose the deviation. Every attack is labeled across the three datasets, and the ground truth is known, so the corpus can be used to train and test anomaly-based IDS techniques.

The dataset has notable limitations: it is small, restricted to a single laboratory training rig and a single industrial protocol (OPC UA), and the released artifacts do not include a documented train/test split or per-class instance counts. Its scenarios are valuable because they target OPC UA and combine network/process behavior with covert physical deviations, but the experimental scope is limited to a laboratory-scale water process with a small attack inventory.

S1.19. ICS-NAD Dataset

In 2026, Zhou et al. released ICS-NAD, a large-scale ICS networking dataset captured from a full-scale, multi-vendor ICS platform built with real industrial equipment at Zhejiang University. Three PLCs are deployed (ABB, Siemens, and Schneider) covering two industrial applications. The thermal-power process is controlled by ABB equipment communicating over a private TCP-based protocol, while the sewage-treatment process is implemented twice in parallel, with Siemens controllers (using S7Comm) and Schneider controllers (using Modbus) operating as alternative configurations for the same plant [46]. The three setups are wired independently, which allows the release to provide separate collections without cross-contamination of traffic. ICS-NAD therefore provides higher-fidelity, multi-vendor traffic from real industrial equipment across several protocols, more representative than the scaled-down laboratory testbeds that dominate the rest of this corpus.

The dataset includes 20 common ICS attack types, falling into four categories: reconnaissance, DoS/DDoS, false data injection (FDI), and man-in-the-middle (MitM). Two attack traffic patterns are defined. The first pattern alternates one minute of normal traffic with four minutes of attack traffic carrying a specific ICS attack. The second pattern is designed to simulate denial-of-service behavior and consists of repeated three-minute cycles with random attack bursts in the second minute, capturing both attack onset and recovery dynamics.

ICS-NAD provides raw packet captures alongside extracted feature sets. Raw traffic is collected via switch-port mirroring and saved in PCAP format; 60 packet- and flow-

based features are then extracted using a modified version of the ICSFlowGenerator workflow originally developed for ICS-Flow [11]. The final release totals 245.96 GB across 272 files, comprising raw PCAPs, labeled feature tables, and timestamp logs used during the labeling pipeline.

Overall, ICS-NAD is a strong dataset covering real-equipment industrial environments, multiple vendor platforms, multiple ICS protocols, broad attack coverage, and labels released alongside both raw and feature-level data.

S1.20. ICS-ADD Dataset

Gaggero et al. (2024) presented the ICS Anomaly Detection Dataset, collected from a laboratory smart-industry ICS testbed assembled to reproduce the architecture of a small operational SCADA deployment. The release combines raw network traffic with the runtime outputs of open-source security-monitoring tools, enabling analysis of both attack behavior and the responses of operational security tools. The simulated physical process is a water-treatment-like scenario involving two tanks that are filled and emptied by pumps. Communication between SCADA (Modbus master) and PLC (Modbus slave) is over Modbus/TCP. The network and security plane comprises a pfSense firewall, a managed switch with a SPAN port for traffic mirroring, OSSIM as the SIEM, and Suricata as the NIDS engine running on OSSIM [47].

The dataset captures a single multi-stage attack scenario structured along the Cyber Kill Chain concept and driven by a compromised internal endpoint that serves as the attacker's pivot. The seven attack stages comprise: DNS tunneling for command-and-control communication; port scanning and Modbus scanning for reconnaissance; SCADA password brute-forcing for credential access; ARP spoofing to establish man-in-the-middle positioning; Modbus false-data injection that causes the pump to activate without operator intervention; and denial of service against the SCADA web service. The released dataset has three components: a raw packet-capture file (traffic_capture_span.pcap), ScadaBR event logs (ScadaBr_events.csv), and OSSIM event logs containing both pfSense firewall records and Suricata alerts [47].

ICS-ADD is one of the few datasets that release security-tool outputs alongside raw network traffic, enabling two evaluation modes simultaneously: training ML-based anomaly detection on the PCAP and comparing ML results against the rule-based decisions produced by the operational IDS/SIEM stack on the same captured traffic.

S1.21. HiTar Dataset

To address the challenge of intrusion detection in smart-manufacturing IIoT environments, Dhaouadi et al. (2025) introduced the HiTar dataset. The dataset is generated using the AREZZO flexible-manufacturing simulator [68], which provides realistic shop-floor scenarios. The simulated environment models a shop-floor automation network with controllers, workstations, RFID reader/writer devices, shuttle stop-and-go elements, and an Ethernet-based Modbus/TCP communication architecture [48].

HiTar is constructed from the LOG files produced by AREZZO and labeled by a script (attack_labelling.sh) released alongside the dataset. The resulting release is a labeled

tabular dataset of 15,842 instances with 39 extracted features per record, including timestamp, source and destination IP, source and destination port, protocol type, TCP-flag indicators, service indicators, and the attack-type label [48].

The dataset contains five labeled classes: Normal, Probing, Remote-to-Local (R2L), User-to-Root (U2R), and Denial of Service (DoS). This taxonomy is analogous to the historical IT-style intrusion-detection datasets such as KDD/NSL-KDD and is not based on OT-specific attack mechanisms such as PLC-logic modification, setpoint alteration, or process-variable falsification. HiTar is therefore suitable for supervised ML-based IDS studies in an IIoT manufacturing scenario, but it is not appropriate for research that requires fine-grained OT-protocol semantics or process-aware attack labels.

S1.22. EDS Dataset

The EDS dataset was created by Xue et al. (2024) as part of a real-time intrusion-detection study based on decision fusion. To address the well-documented simulation-to-reality gap in ICS cybersecurity datasets, the authors built a full-hardware, high-fidelity Ethanol Distillation System (EDS) testbed. The platform realizes a scaled-down but fully functional ethanol-water distillation process capable of both cold start-up and normal operation.

Communication is implemented over Ethernet between a Siemens PLC and the supervisory computer using the Siemens S7Comm protocol. Data acquisition is performed by a Python program that uses the snap7 library to periodically poll the PLC, recording both the values of the monitored PLC data points and the associated network communication metadata. The release contains 72,965 data points captured over 10 hours of normal operation; attack-scenario recordings bring the full released corpus to 843,321 instances as reported in Table 7. Each record carries 47 parameters: one timestamp, eight control parameters, 24 digital parameters, and 14 analog parameters [49].

The attack design covers seven ICS-specific threat categories: information leakage, replay attack, command injection, sensor data tampering, control parameter tampering, multi-point attack, and physical attack. The seven attacks are organized along an escalating attacker-capability ladder: from network access alone, to knowledge of the physical process and control logic, to direct access to field devices. This makes EDS richer than purely network-based IDS releases, since it captures cyber and physical-process characteristics in the same experimental environment. The released dataset does not provide complete per-class label distributions for each attack type.

S1.23. HAI Dataset

Shin et al. (2020, 2021) introduced the HIL-based Augmented ICS (HAI) Security Dataset to address the scarcity of cyber-physical-system datasets with strong cross-process coupling and repeatable attack execution. HAI was produced at the Affiliated Institute of ETRI (South Korea) by combining three independently developed laboratory-scale testbeds (a GE turbine testbed, an Emerson boiler testbed, and a FESTO modular production system water-treatment testbed) via a dSPACE hardware-in-the-loop (HIL) simulator that emulates steam-turbine power generation and pumped-storage hydropower

generation. Data are collected through an OPC UA gateway that interfaces with Siemens PLCs and ET200 remote I/O modules [50,51]. The dataset is publicly available at https://github.com/icsdataset/hai with a separate technical manual.

The testbed exposes four process areas: a boiler process (P1) on Emerson Ovation DCS, a turbine process (P2) on GE Mark VIe DCS, a water-treatment process (P3) on a Siemens S7-300 PLC, and an HIL simulation (P4) that synchronises the boiler and turbine processes with a virtual steam-turbine power-generation model and drives the P3 water-treatment pump and valve via a pumped-storage hydropower model. The cross-process coupling supplied by the HIL simulator is HAI's principal differentiator from single-process ICS datasets such as SWaT or BATADAL.

The HAI family has had four releases: HAI 1.0 / 20.07 (February and August 2020; 38 attack scenarios, 59 monitored SCADA tags), HAI 21.03 (2021; 50 attack scenarios, 78 tags), HAI 22.04 (2022; 58 attack scenarios, 86 tags, with detection difficulty approximately four times higher than HAI 21.03 per the official documentation), and HAI 23.05 (2023; 52 attack scenarios, 86 tags) with the companion HAIEnd 23.05 (225 internal boiler-DCS tag values intended for endpoint-threat-detection research).

Each release provides CSV files with a one-second-resolution timestamp column, SCADA tag values, and a final attack-label column. From HAI 22.04 onward, per-process attack labels (attack_P1, attack_P2, attack_P3) were replaced by per-attack target metadata identifying which process each attack manipulated. Attacks are deliberately injected by an automated attack tool that manipulates feedback-control loop components (set-points, controller parameters, sensor readings, and actuator commands) at the SCADA-point level rather than the network-protocol level. The HAI 22.04 release in particular is designed for evaluating attacks with process-level impact but no obvious network-level signatures, which makes HAI complementary to network-IDS-oriented datasets such as Electra and ICS-NAD.

Multi-version documentation, the eTaPR evaluation library, and public HAICon competition baselines together make HAI one of the most reusable datasets in the corpus. Its principal limitation is that the main releases provide process-level CSV time-series only and do not include raw PCAP files.

Table S1. Dataset availability and access.

Availability status, access link or request page, and access date for the 23 datasets reviewed. Datasets were accessed in May 2026; availability and links were re-verified in June 2026. Public datasets give a direct repository or archive link; Request/Restricted datasets give a request/landing page or an access note.

Sr	Dataset	Availability	Access link / request page
01	SWaT	Request	https://itrust.sutd.edu.sg/itrust-labs_datasets/ (request)
02	WADI	Request	https://itrust.sutd.edu.sg/itrust-labs_datasets/ (request)
03	EPIC	Request	https://itrust.sutd.edu.sg/itrust-labs_datasets/ (request)
04	BATADAL	Public	https://www.batadal.net/
05	S317	Request	https://itrust.sutd.edu.sg/itrust-labs_datasets/ (request)
06	MSU-GP	Public	https://sites.google.com/a/uah.edu/tommy-morris-uah/ics-data-sets
07	MSU-PWR	Public	https://sites.google.com/a/uah.edu/tommy-morris-uah/ics-data-sets
08	ICS-Flow	Public	https://www.kaggle.com/datasets/alirezadehlaghi/icssim
09	Lemay	Public	https://github.com/antoine-lemay/Modbus_dataset
10	Electra	Restricted	On request from the authors
11	Rodofile	Public	https://github.com/qut-infosec/2017QUT_S7comm
12	WUSTL-IIoT	Public	https://www.cse.wustl.edu/~jain/iiot2/index.html
13	Edge-IIoTset	Public	https://www.kaggle.com/datasets/mohamedamineferrag/edgeiiotset-cyber-security-dataset-of-iot-iiot
14	HIL-WDT	Public	https://ieee-dataport.org/open-access/hardware-loop-water-distribution-testbed-wdt-dataset-cyber-physical-security-testing
15	X-IIoTID	Public	https://ieee-dataport.org/documents/x-iiotid-connectivity-and-device-agnostic-intrusion-dataset-industrial-internet-things
16	TEP	Public	https://doi.org/10.7910/DVN/6C3JR1 (Harvard Dataverse)
17	TLIGHT	Restricted	On request from the authors
18	IUNO	Restricted	On request from the authors
19	ICS-NAD	Public	https://www.scidb.cn/en/detail?dataSetId=380298d0714740dd91413b5db6305dfd
20	ICS-ADD	Public	https://ieee-dataport.org/documents/ics-add-smart-industry-testbed-dataset-cyber-physical-security-monitoring-testing
21	HiTar	Request	On request from the authors
22	EDS	Public	https://github.com/Margaux2022/Cyber-security-dataset-of-EDS
23	HAI	Public	https://github.com/icsdataset/hai
